# Supplementary material for: Short single-stranded DNAs with putative non-canonical structures comprise a new class of plasma cell-free DNA
Source: BMC Biol. 2021 Oct 14;19:225. doi: 10.1186/s12915-021-01160-8 (PMC8518174; doi:10.1186/s12915-021-01160-8)
Supplement: Supplementary file 1 — Additional file 1: Figures S1-S18, Tables S1-2, S6-11, Supplementary methods, Supplementary Information. Figure S1. – Short single-stranded DNA in cell-free blood fractions. Figure S2. – The membranous fraction of the plasma does not contain cfDNA. Figure S3. – TACS-T4 scheme for the library preparation from ssDNA. Figure S4. – The size distributions of cfDNA fragments in the libraries prepared with different pretreatments reflect different forms of DNA. Figure S5. – Two major clusters of cfDNA were consistently detected with the three ssDNA-adapted library preparation methods. Figure S6. – Locations of the C3D peaks are well conserved among individuals. Figure S7. – C3D is enriched in the regulatory regions of genes. Figure S8. – The short single-stranded cfDNA detected by other studies is enriched in the open chromatin regions and TFBS. Figure S9. – The colocalization of C3D peaks with TFBS. Figure S10. – The colocalization of short single-stranded cfDNA with TFBS based on the literature. Figure S11. – The colocalization of C3Don and C3Doff reads and short single-stranded cfDNA with the open chromatin regions and TFBS based on the literature. Figure S12. – The base composition of C3D peaks was highly biased. Figure S13. – The antisense strand of G4-Seq reads well localize on the C3D peaks. Figure S14. – Colocalization analysis of the G4 structure with short single-stranded cfDNA in the literature as well as C3Don and C3Doff reads. Figure S15. – Enriched C3D reads on the G4 structure with different library preparation methods. Figure S16. – C3D peaks without antisense G4 structures are less enriched in the regulatory regions. Figure S17. – Colocalization of C3D peaks and repetitive sequences. Figure S18. – The antisense strands of G4 motifs are enriched at the 5′-end of C3D peaks. Table S1. – Summary of the library preparations and sequencing methods Table S2. – The number of reads and peaks calculated from two publicly available single-stranded cfDNA datasets Tabl [file 12915_2021_1160_MOESM1_ESM.pdf]

Additional file 1

Short single-stranded DNA with putative non-canonical structures comprise a novel class of plasma cell-free DNA

Osamu Hisano, Takashi Ito*, and Fumihito Miura*

Department of Biochemistry, Kyushu University Graduate School of Medical Sciences, 3-1-1 Maidashi, Higashi-Ku, Fukuoka 812-8582, Japan

* To whom correspondence should be addressed. Tel: +81-92-642-6100; Fax: +81-92-642-6203; Email: fumihito@med.kyushu-u.ac.jp

* Correspondence may also be addressed to Takashi Ito. Tel: +81 92 642 6095; Fax: +81 92 642 6203; Email: tito@med.kyushu-u.ac.jp

Supplementary Figures

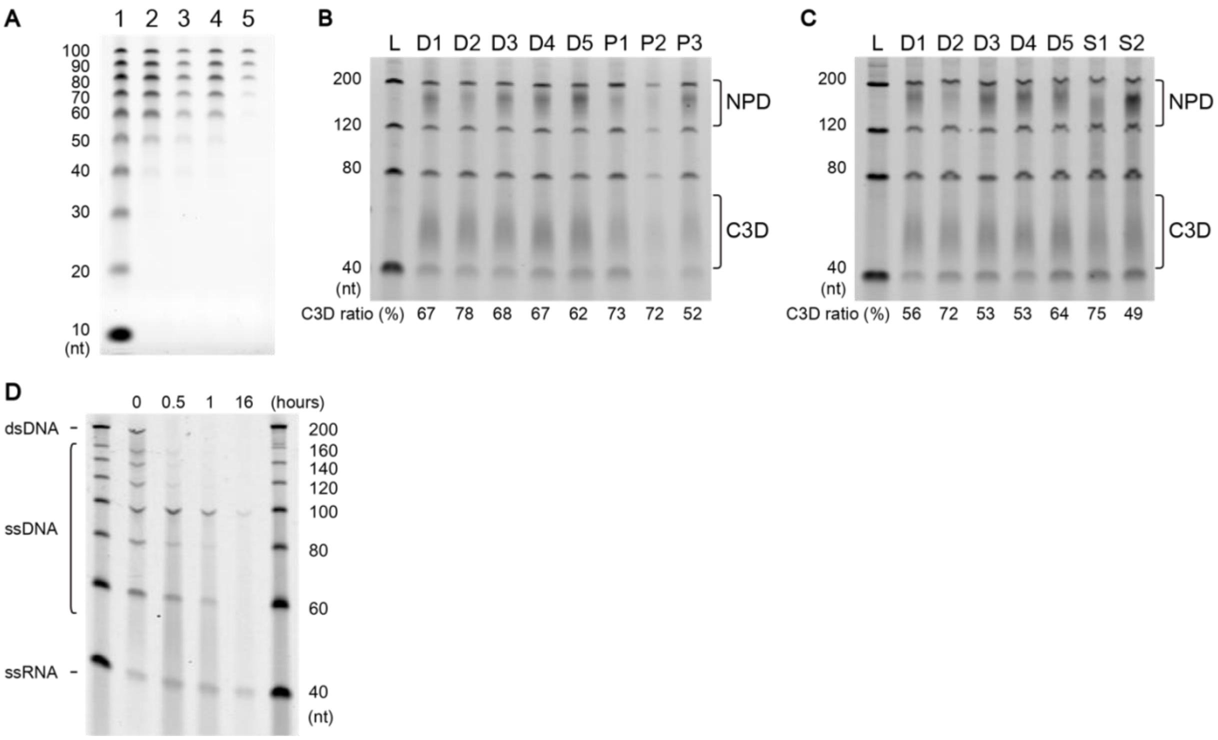

Figure S1. Short single-stranded DNA in cell-free blood fractions.

A. DNA samples recovered using four commercially available kits were compared. As a model, the 10 bp DNA step ladder from Promega (Fitchburg, WI, USA) was used (lane 1). The kits used were the QIAamp Circulating Nucleic Acid Kit (Qiagen, Hilden, Germany) (lane 2), the Plasma/Serum Cell-Free Circulating DNA Purification Mini Kit from Norgen Biotek (Thorold, Canada) (lane 3), the NucleoSpin Plasma XS from Takara Bio Inc. (Shiga, Japan) (lane 4), and the NEXTprep-Mag cfDNA Isolation Kit from PerkinElmer (Waltham, MA, USA) (lane 5).

B and C. The cfDNAs purified from plasma (B) and serum (C) were compared. Plasma and serum were obtained from five healthy donors and commercial sources (see Table S7). For details of the experimental procedures, see Supplementary Methods.

D. DNase I treatment was performed over an extended period, as shown in Fig. 1G.

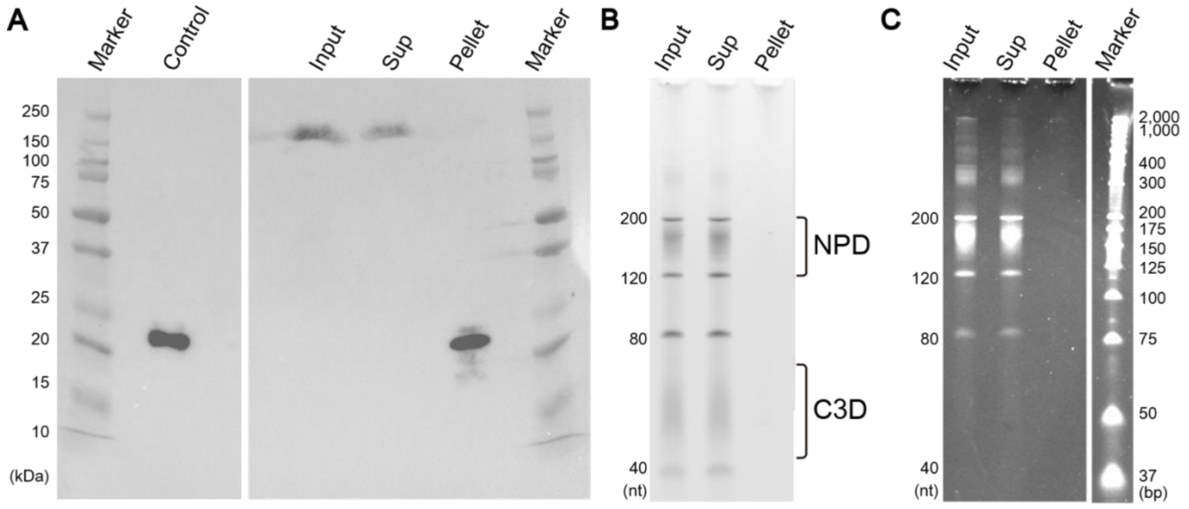

Figure S2. The membranous fraction of the plasma does not contain cfDNA.

The plasma from a healthy individual (Donor 1) was fractionated via ultracentrifugation. The supernatant and pellet were used for western blotting and cfDNA purification.

A. Western blotting was performed using an antibody raised against the exosome specific protein marker CD9. As a positive control, purified exosomes from COLO201 cells (FUJIFILM Wako, Osaka, Japan) were loaded (Control). The loading amounts of the plasma before ultracentrifugation (Input) and the supernatant after separation (Sup) were equivalent, whereas 20 times the equivalent amount was loaded for the pellet (Pellet).

B. The cfDNAs purified from the plasma (Input), supernatant (Sup), and pellet (Pellet) after ultracentrifugation were analyzed by denaturing gel electrophoresis. As an internal control, the oligonucleotide mixture (see Supplementary Methods) was spiked into the plasma before centrifugation. After purification, cfDNA was fluorescently labeled and loaded onto the gel. As in A, the input (Input) and the supernatant (Sup) loading amounts were equivalent, whereas 20 times the equivalent amount was loaded for the pellet (Pellet).

C. The gel analyzed in B was stained with SYBR Gold nucleic acid gel stain (ThermoFisher Scientific, Waltham, MA, USA).

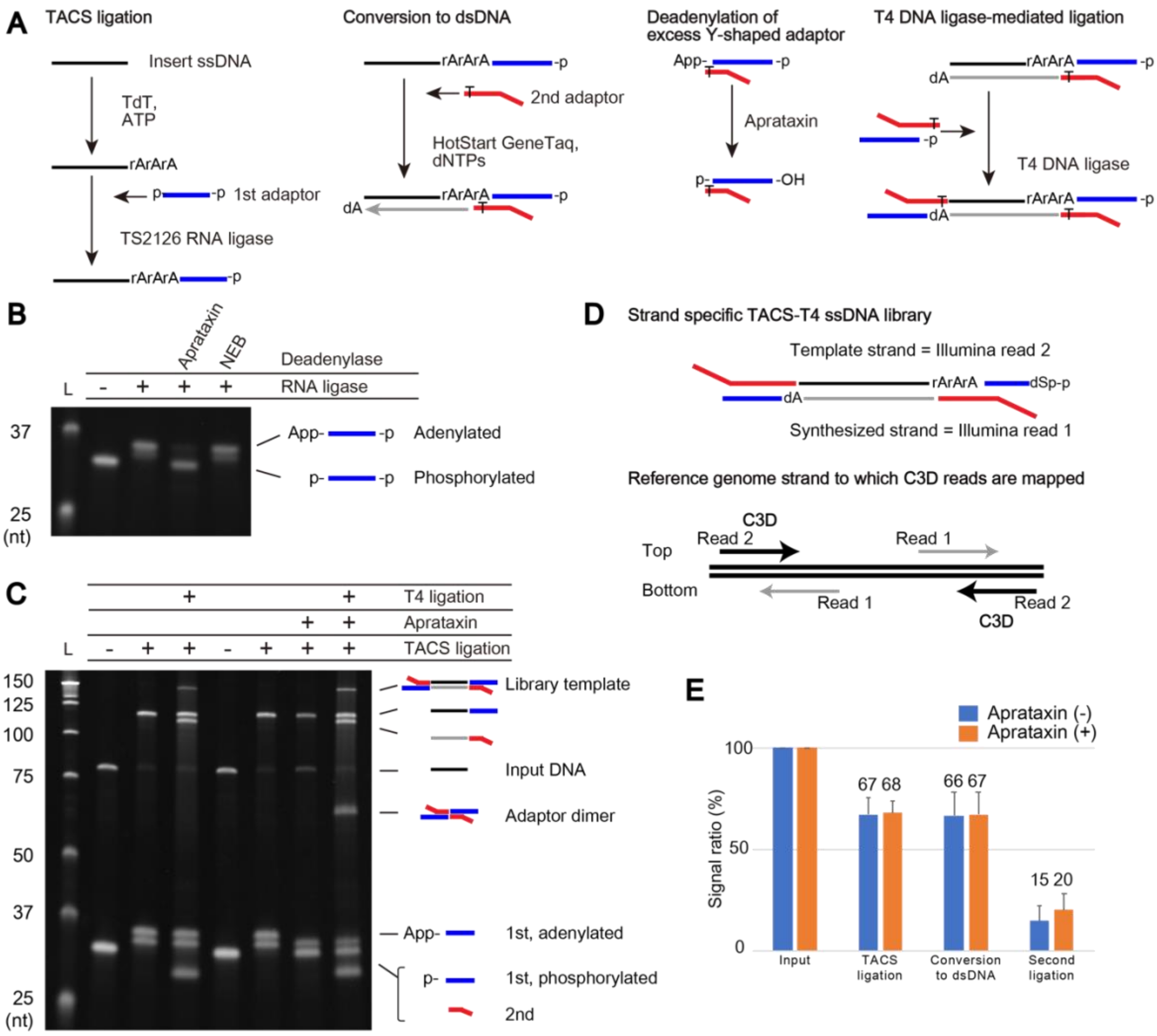

Figure S3. TACS-T4 scheme for the library preparation from ssDNA.

A. The details of each step of the TACS-T4 scheme.

B. Aprataxin-mediated deadenylation of the adaptor. The TS2126 RNA ligase used in TACS ligation adenylates the 5′-phosphorylated end of the adaptor. Since the 3′-end of the adaptor is also phosphorylated to repress its concatenation (see Table S8 for the structure of the oligonucleotide used) and the RNA ligase also adenylates the 3′-phosphorylated end of the adaptor, two bands are seen after the RNA ligase reaction. It seems that the 5′ adenylation inhibits T4 DNA ligase-mediated ligation. Therefore, an efficient method to remove the adenylates was required. We found that recombinant human aprataxin is suitable for removing the adenylates in the solution following TACS ligation. Although 5' deadenylase is available from New England Biolabs (NEB; Ipswich, MA, USA), this enzyme seems to remove adenylates in the solution following TACS ligation poorly.

C. The efficiencies of adaptor tagging with and without aprataxin were compared. The enzyme enhances the tagging of the second adaptor with T4 ligase.

D. The strand specificity of the TACS-T4 scheme. As the TACS ligation product contains a few adenylates, if a DNA polymerase without reverse transcriptase activity is used, PCR amplification will occur only from the strand without the adenylates. Based on the design of the adaptor, read one and read two correspond to the reverse strand and the forward strand of target DNA, respectively.

E. The amounts of products from each step in the TACS-T4 scheme were calculated based on electrophoresis signals relative to the input DNA signal. Averages and standard deviations of three replicates are shown. A representative image is shown in Fig. S3C.

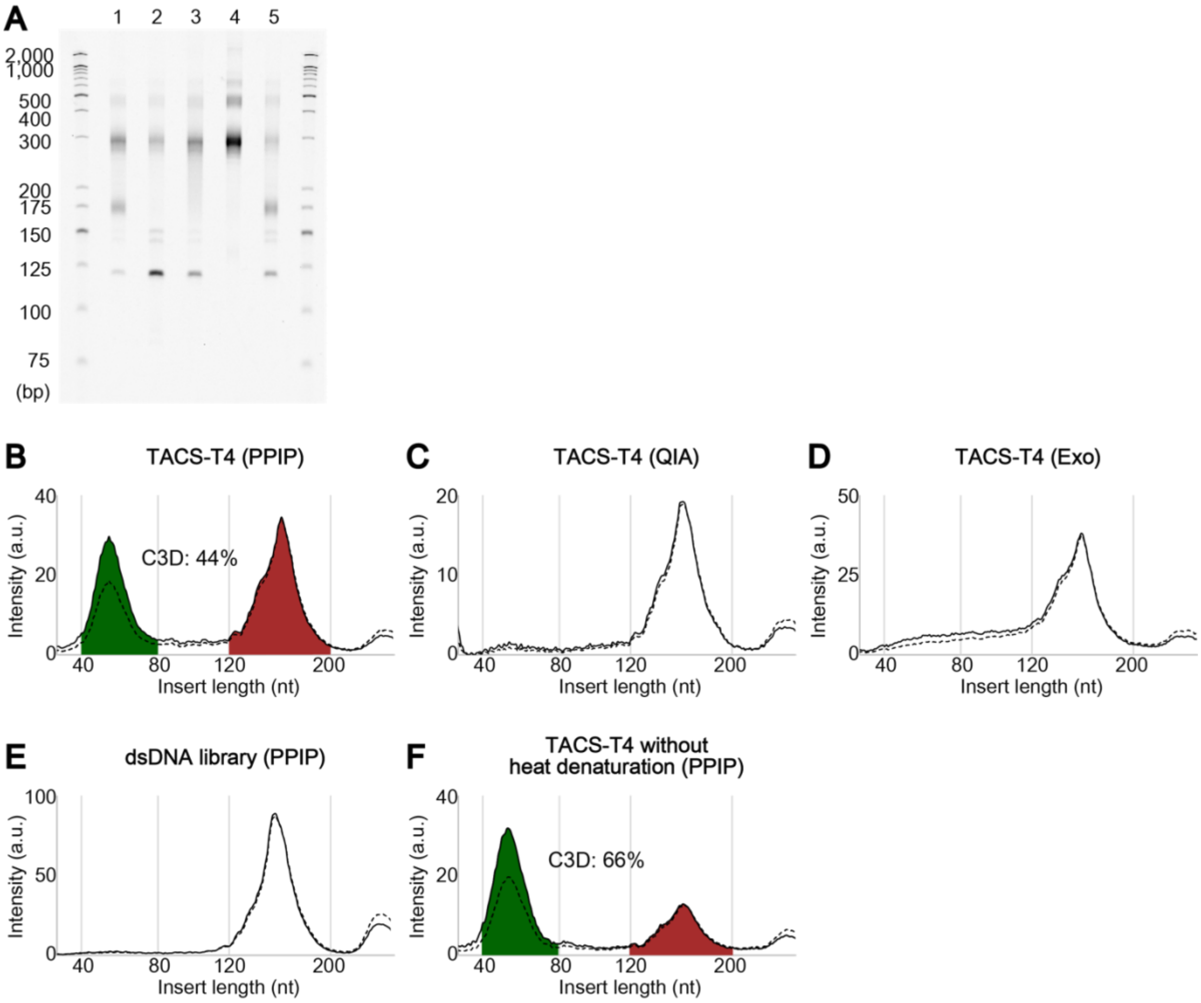

Figure S4. The size distributions of cfDNA fragments in the libraries prepared with different pretreatments reflect different forms of DNA.

A. The gel image from denaturing gel electrophoresis of amplified libraries with real-time PCR quantification is shown. The gel was stained with SYBR Gold nucleic acid gel stain (ThermoFisher Scientific). The loaded libraries were prepared from cfDNA purified using PPIP (lanes 1, 3, 4, and 5) or QIAamp Circulating Nucleic Acid Kit (Qiagen) (lane 2). For lane 2, exonuclease treatment was performed before the cfDNA purification. The libraries were prepared using the TACS-T4 scheme (lanes 1, 2, 3, and 5) or ThruPLEX DNA-Seq kit (Takara Bio Inc.) (lane 4). For lanes 1–3, the cfDNAs were heat-denatured before the TACS-T4 scheme. Note that the libraries shown here are the same as the ones shown in Fig. 2C–G. B–F. The electropherograms of lanes 1 (B), 2 (C), 3 (D), 4 (E), and 5 (F) of the gel image in A. The signal intensity of the raw data (dashed lines) and after normalization with the molecular weight of the DNA (solid lines) are shown. The areas of C3D (green, 40–80 nt) and NPD (red, 120–200 nt) were calculated, and the relative fraction of C3D is indicated for B–F. a.u.; arbitrary unit.

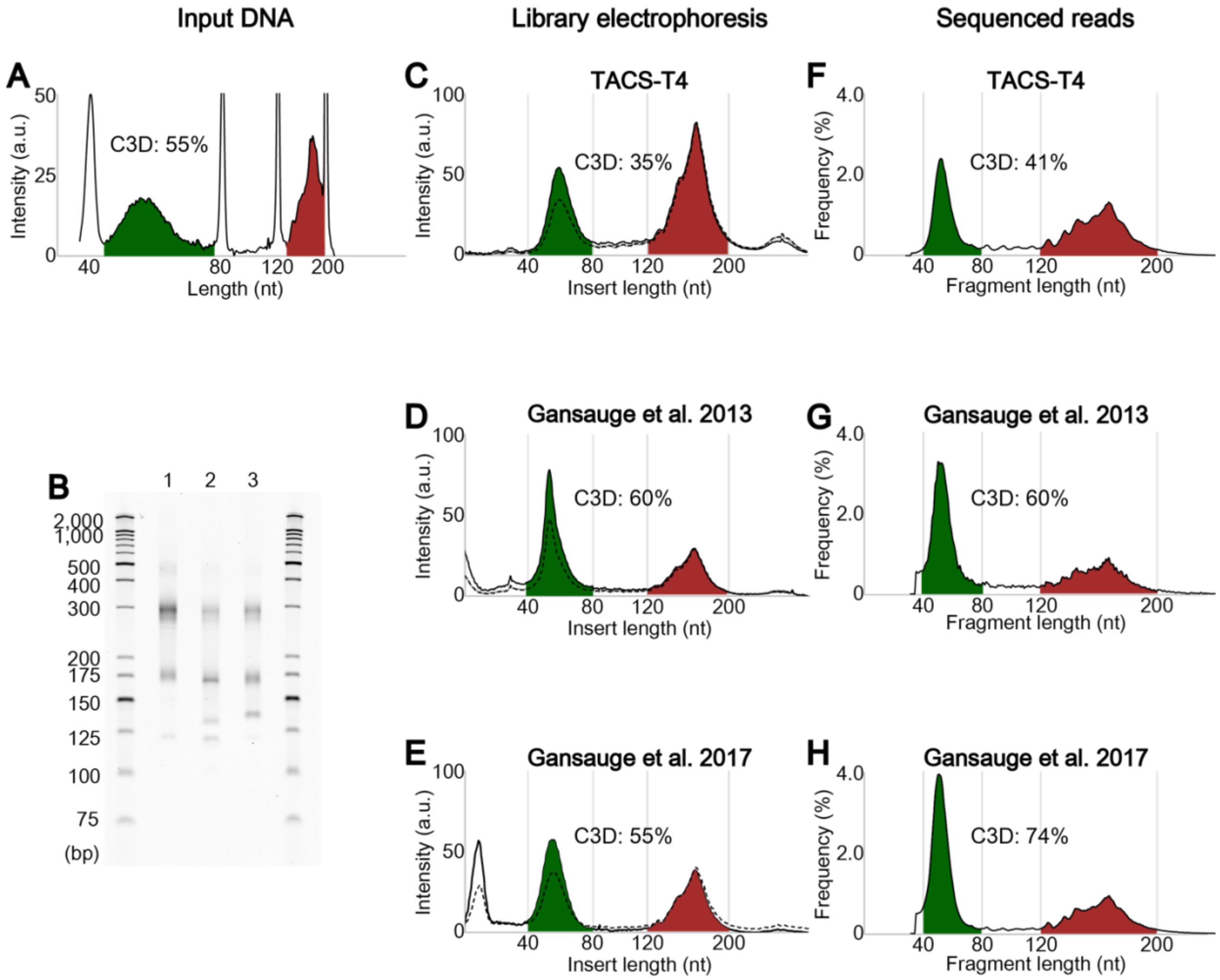

Figure S5. Two major clusters of cfDNA were consistently detected with the three ssDNA-adapted library preparation methods.

A. The electropherogram of input cfDNA purified with the PPIP method, same as Fig. 1D.

B. The gel image from denaturing gel electrophoresis of amplified libraries after real-time PCR quantification is shown. The libraries were prepared using the TACS-T4 scheme (lane 1), ssDNA-adapted protocol by Gansauge et al. (2013) (20) (lane 2), or Gansauge et al. (2017) (21) (lane 3). The gel was stained with SYBR Gold nucleic acid gel stain (ThermoFisher Scientific).

C–E. The electropherograms of lanes 1 (C), 2 (D), and 3 (E) of the gel image in B. The signal intensity of the raw data (dashed lines) and after normalization with the molecular weight of the DNA (solid lines) are shown.

F–H. The size distribution of sequenced reads for libraries, same as lanes 1 (F), 2 (G), and 3 (H) are shown.

The areas of C3D (green, 40–80 nt) and NPD (red, 120–200 nt) were calculated, and the relative fraction of C3D is indicated for C–H. a.u.; arbitrary unit

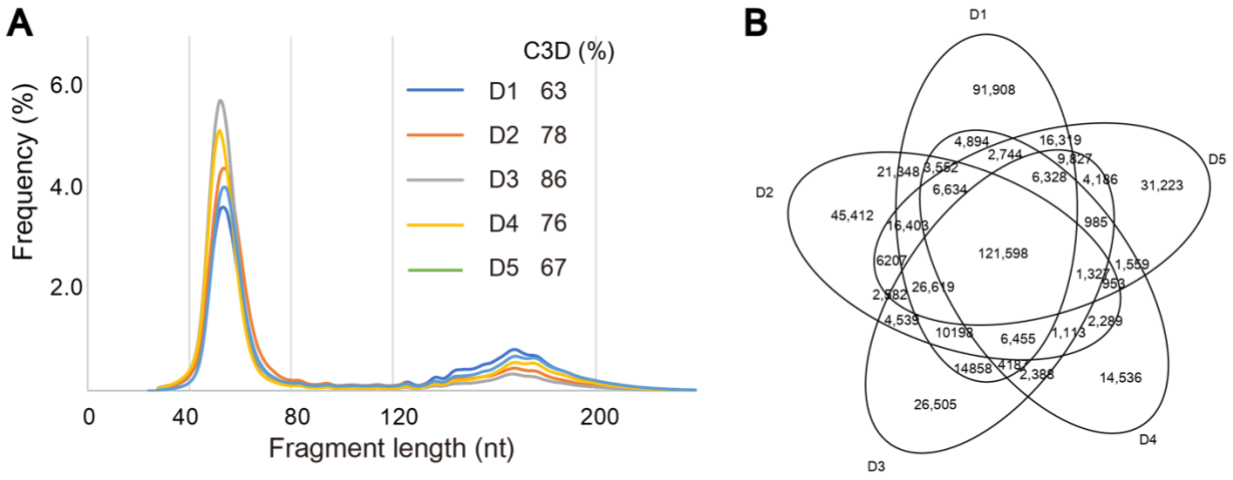

Figure S6. Locations of the C3D peaks are well conserved among individuals.

A. The size distributions of cfDNA fragments detected by sequencing for five individuals are overlaid. The relative amount of C3D reads are calculated and indicated for each sample.

B. Venn diagram showing overlaps of C3D peaks among the five donors.

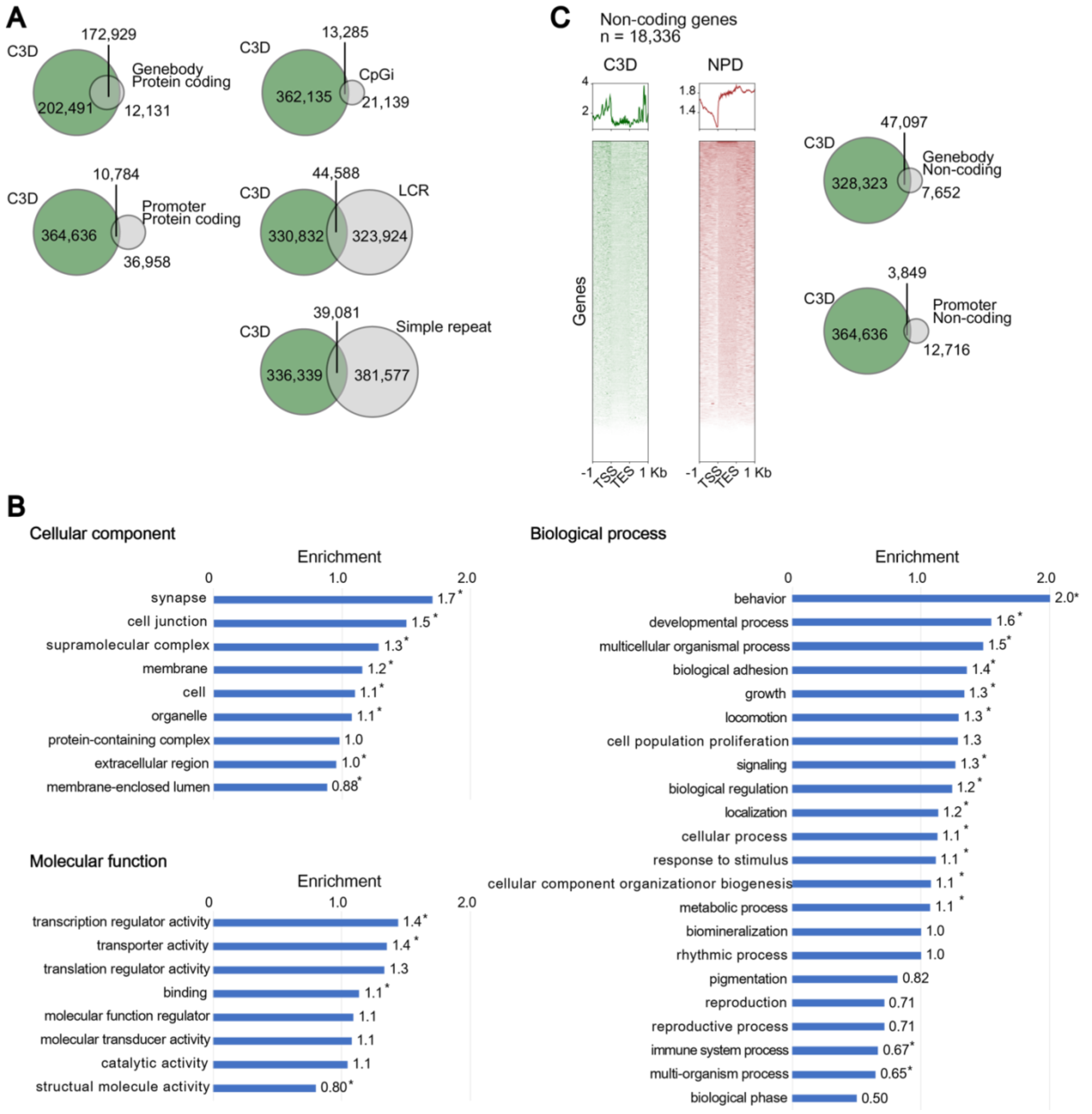

(Continues on the next page)

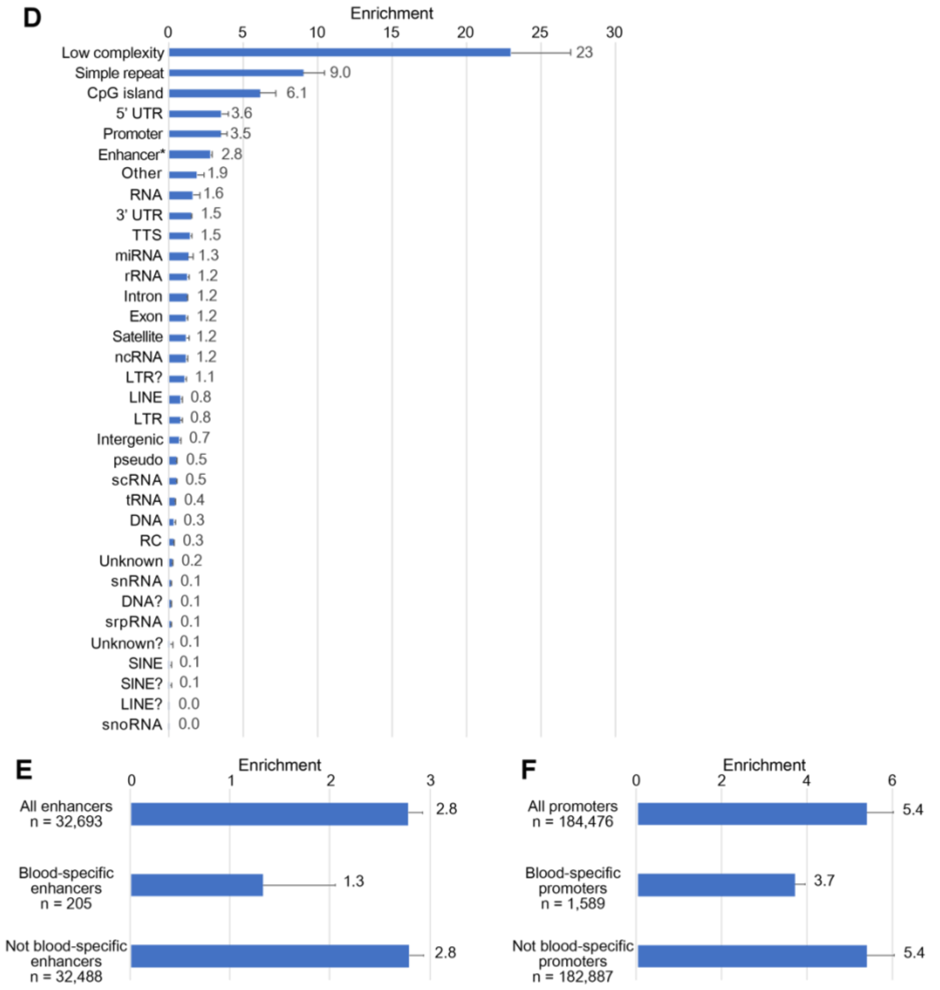

Figure S7. C3D is enriched in the regulatory regions of genes.

A. The colocalization of C3D peaks and the annotations enriched in the C3D peaks are shown in Venn diagrams.

B. Fold enrichment of gene ontology (GO) terms for genes that contain C3D peaks in their promoters or 5′ UTRs (7,634) compared to that for all reference genes (n = 20,851). *p-value < 0.05 (Fisher’s exact test).

C. Aggregation plots of the normalized read coverages of C3D and NPD around the non-coding genes are shown.

D. The enrichment of C3D peaks was calculated for each annotation provided by the “Detailed Annotation” of HOMER annotatePeaks. For enhancers, the data downloaded from the FANTOM5 project (27, 28) was used.

E and F. The blood specificity from the enriched C3D peaks in enhancers (E) and promoters (F) was evaluated using the dataset downloaded from the FANTOM5 project (27, 28). The blood specificity of enhancers and promoters was defined when the CAGE signal detected for the blood cells exceeded 90% of the total CAGE signal assigned.

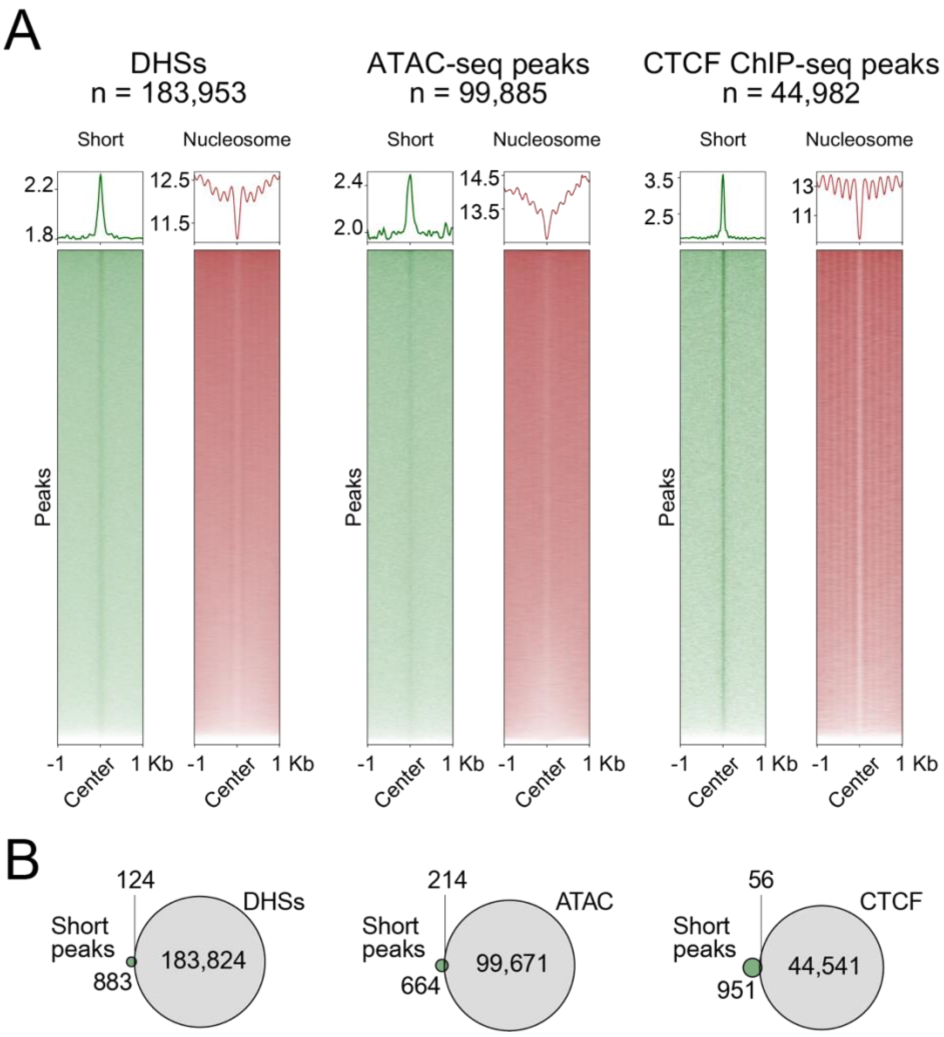

(Continues on the next page)

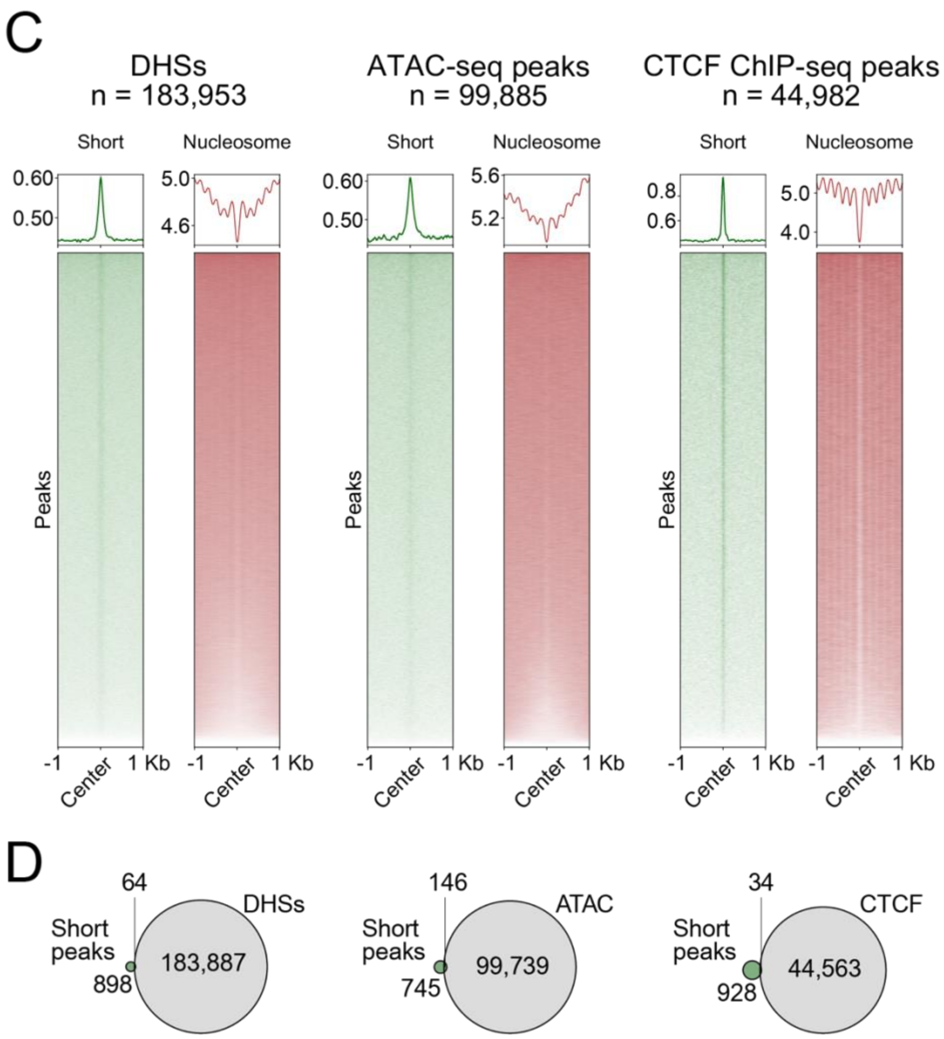

Figure S8. The short single-stranded cfDNA detected by other studies is enriched in the open chromatin regions and TFBS.

A and C. Aggregation plots of the short (35–80 nt) and nucleosome size range (120–180 nt) single-stranded cfDNA reads presented by Snyder et al. (15) (15) (A) and Burnham et al. (23) (C) on DNaseI-hypersensitive sites (DHSs), the peaks of ATAC-Seq (ATAC), and CTCF binding sites (CTCF).

B and D. The overlapping of the peaks of single-stranded cfDNA ranging from 35 to 80 nt (cfDNA peaks) determined by Snyder et al. (15) (B) and Burnham et al. (23) (D) with DHSs, the peaks of ATAC-Seq, and CTCF binding sites.

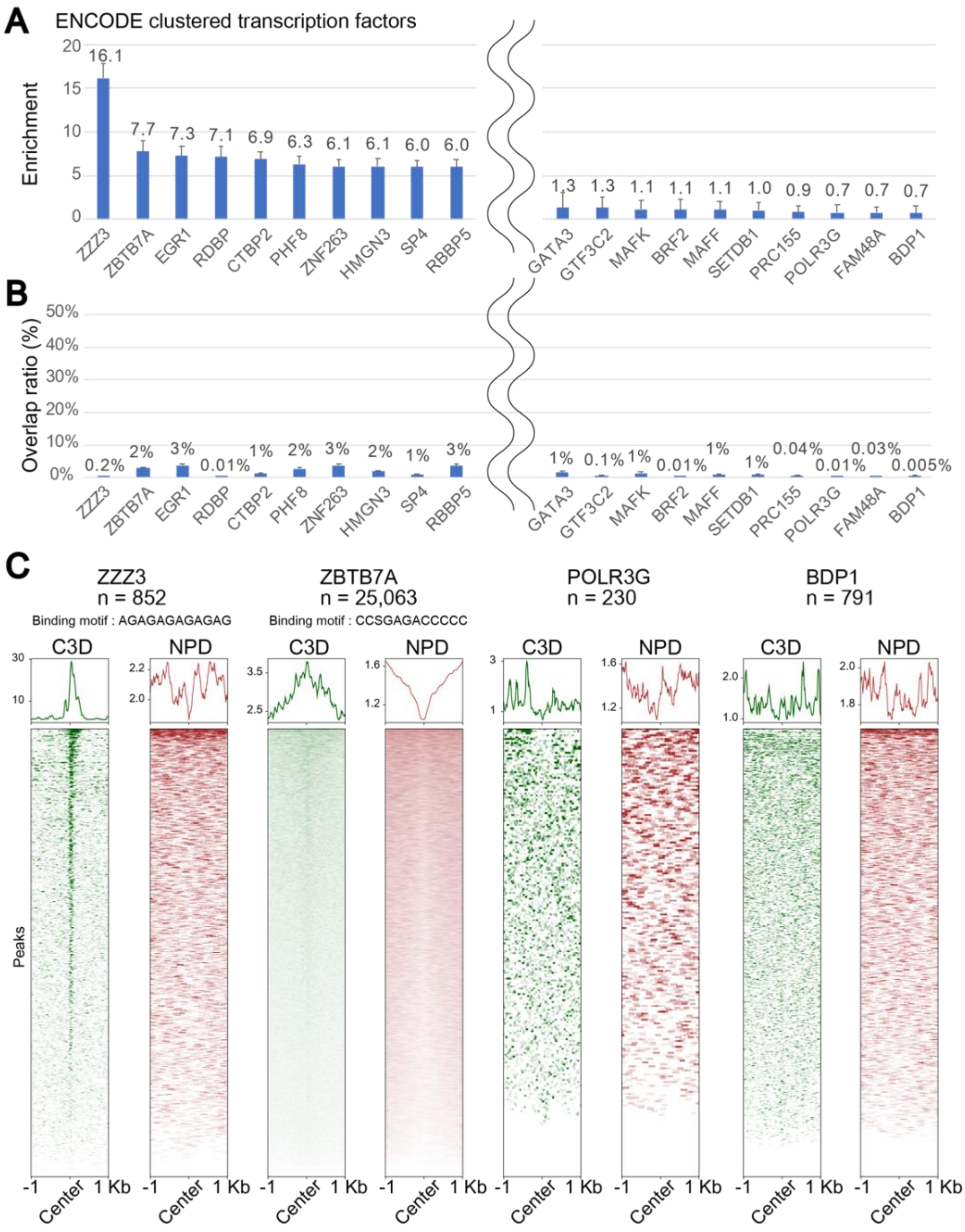

(Continues on the next page)

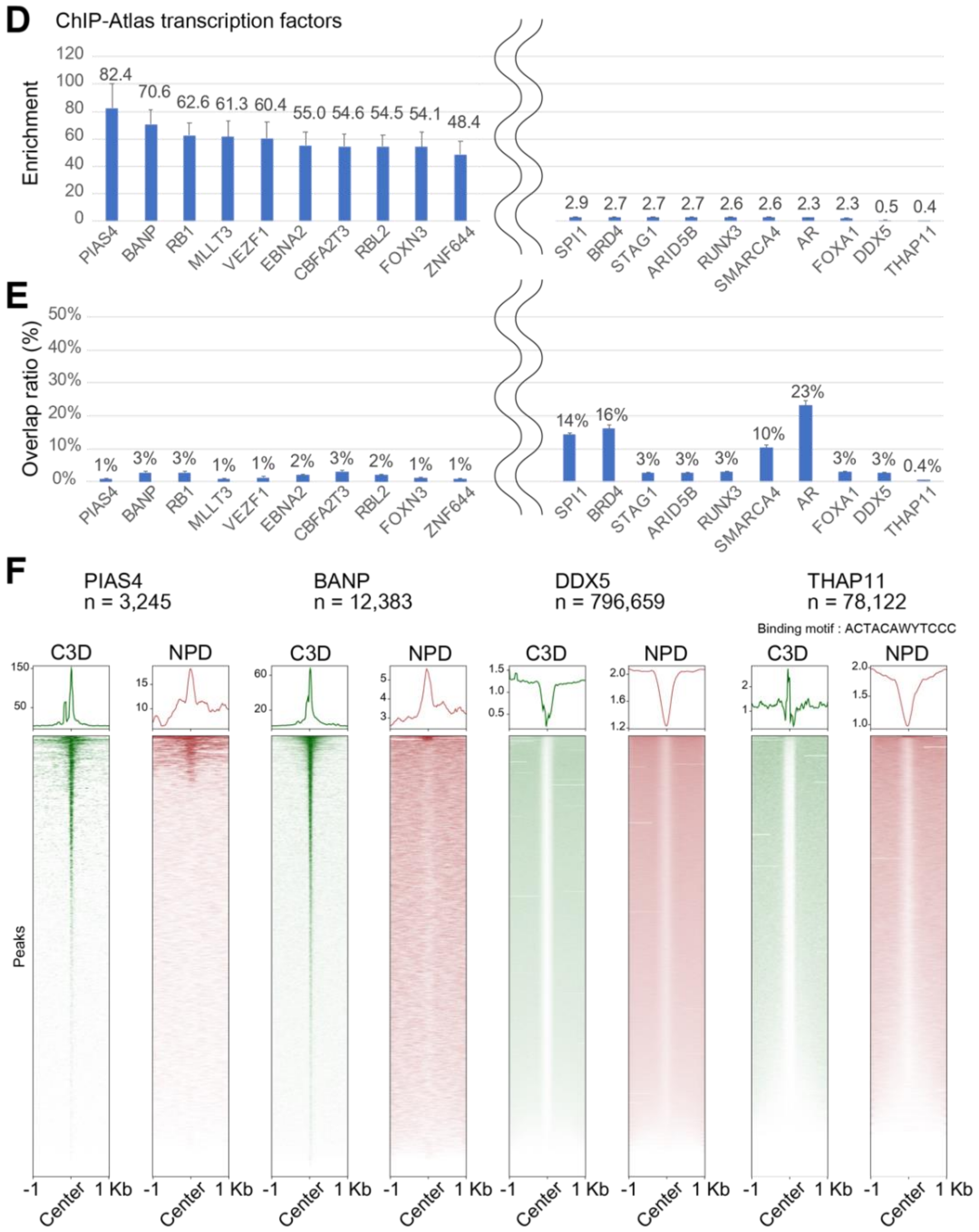

Figure S9. The colocalization of C3D peaks with TFBS.

The colocalizations of C3D peaks with TFBSs defined by the ENCODE project (A–C) and ChIP-Atlas (D–F) are shown.

A and D. The relative enrichment of C3D peaks on the binding sites of individual TFs over the entire genome was calculated.

B and E. The fractions of the C3D peaks overlapped with those of the individual TFBS.

C and F. Examples of aggregation plots of C3D and NPD reads around the binding sites of individual TFs are shown. Consensus-binding motifs were obtained from ChIPBase (29).

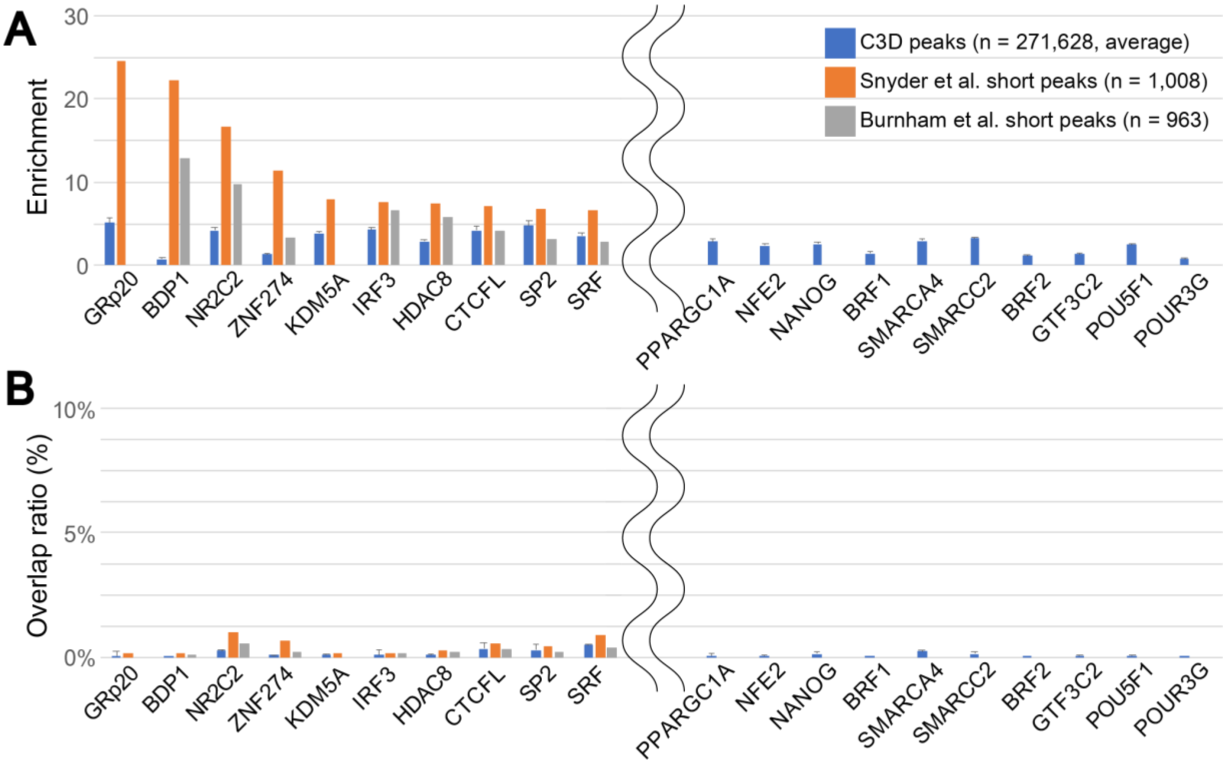

Figure S10. The colocalization of short single-stranded cfDNA with TFBS based on the literature.

The colocalization of C3D peaks (same as S9), the peaks of the short single-stranded cfDNA (35–80 nt) identified by Snyder et al. (15) and Burnham et al. (23) with TFBSs defined by the ENCODE project are shown.

A. The relative enrichment of the peaks on the binding sites of individual TFs over the entire genome was calculated.

B. The fractions of peaks that overlapped with those of individual TFBS.

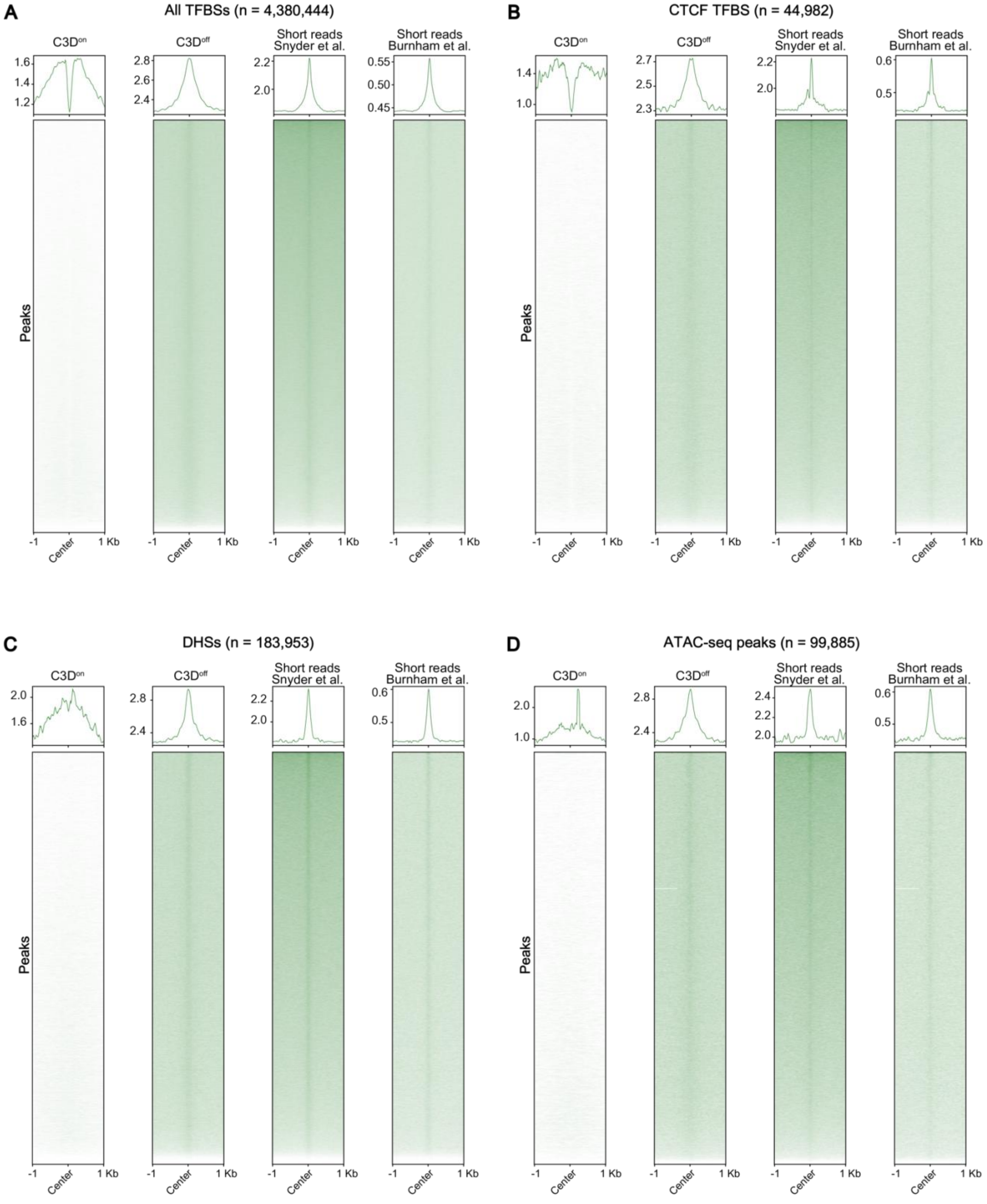

(Continues on the next page)

Figure S11. The colocalization of C3D^{on} and C3D^{off} reads and short single-stranded cfDNA with the open chromatin regions and TFBS based on the literature.

Aggregation plots of C3D^{on} and C3D^{off} reads and short (35–80 nt) cfDNA identified in Snyder et al. (15) and Burnham et al. (23) on all of the transcription factor binding sites (A), CTCF binding sites (B), DHSs (C), and the peaks of ATAC-seq (D) are shown. For all the plots, the TFBS peaks were sorted according to the read number of short cfDNA fragments in Snyder et al. (15).

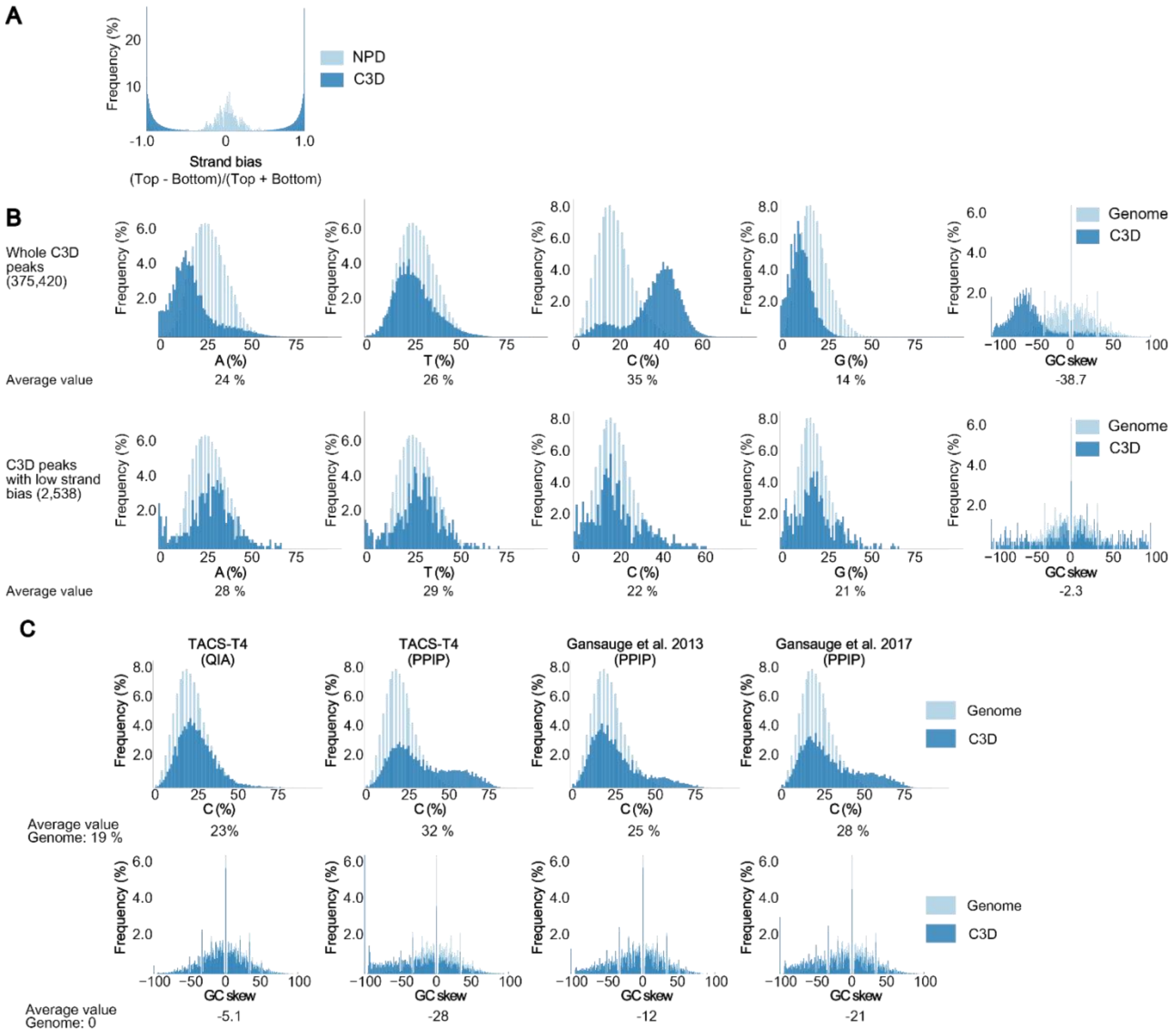

Figure S12. The base composition of C3D peaks was highly biased.

A. C3D reads are mapped with strong strand biases. The alignments of C3D and NPD reads mapped on the top and bottom strands of the reference genome were served for peak calling by MACS2 separately. Then, the normalized read coverages of the top and bottom strands were calculated for each of the peaks. The strand biases were calculated with the formula at the bottom of the histogram.

B. Histograms display the base composition of C3D peaks (blue) and the randomly chosen 50-mers from the reference genome (light blue). The peaks identified by MACS2 with the reads mapped on the top and bottom strands of the reference genome separately were used for the calculation of the base composition. The distributions of all C3D peaks (top) and peaks

with low strand bias (bottom) are shown. If 50% of peak coordinates overlapped with a peak on the opposite strand, the peak was assumed to have less strand bias.

C. Histograms of the C-content and GC-skew for the reads (blue) and randomly chosen 50-mers from the reference genome (light blue). Three different library preparation methods including TACS-T4, Gansuage et al. (2013) (20), and Gausuage et al. (2017) (21) are compared for the short reads (35–75 bp). As a control, a library prepared with TACS-T4 library from cfDNA purified with the QIAamp Circulating Nucleic Acid Kit (Qiagen) was also calculated.

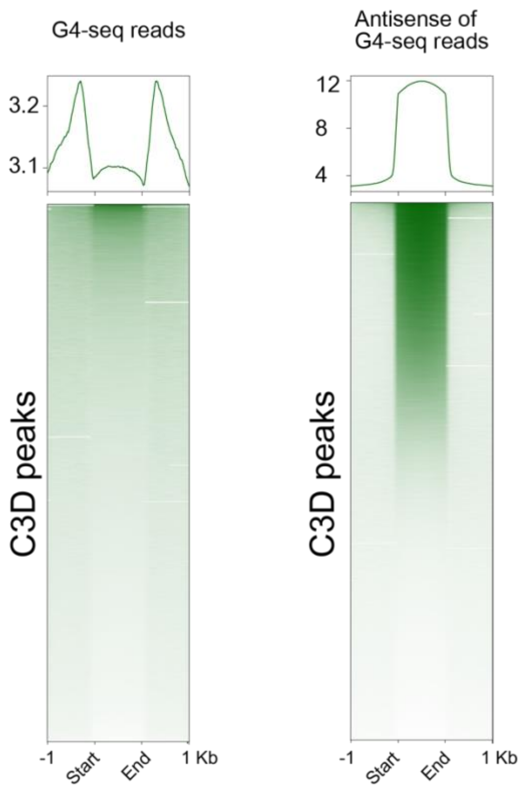

Figure S13. The antisense strand of G4-Seq reads well localize on the C3D peaks. Aggregation plots of G4-seq read coverage of the C3D peaks. The normalized read coverage of G4-seq (left) and that of the opposite strand (right) are shown.

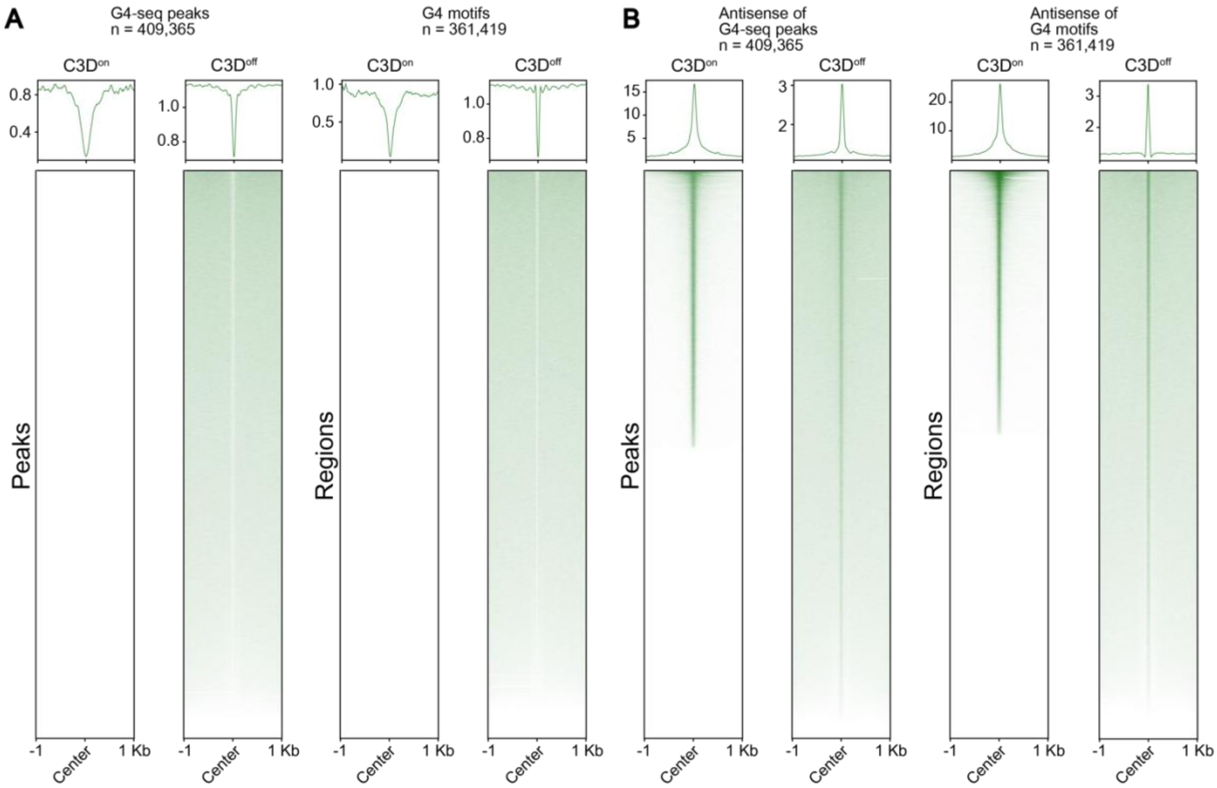

C

	G4-positive	G4-negative
C3Don	25,504,128 (8.9%)	43,868,156 (15%)
C3Doff	8,292,426 (2.9%)	209,531,056 (73%)

p-value < 0.01

(Continues on the next page)

Figure S14. Colocalization analysis of the G4 structure with short single-stranded cfDNA in the literature as well as C3D^{on} and C3D^{off} reads.

A and B. Aggregation plots of C3D^{on} and C3D^{off} reads on the same (A) and antisense (B) strands of G4-seq peaks (left) and putative G4 sequences defined by the *quadparser* algorithm (right).

C. Numbers and fractions of C3D^{on} and C3D^{off} reads overlapping with either G4-seq peaks or the G4 motif (G4-positive reads), and those not overlapping either (G4-negative reads) are shown. Statistical analysis was performed using Fisher's exact test.

D and E. Aggregation plots of short (35–80 nt) single-stranded cfDNA reads presented by Snyder et al. (15) and Burnham et al. (23) on the same (D) and antisense (E) strands of G4-seq peaks (left) and putative G4 sequences defined by the *quadparser* algorithm (right).

F and G. Venn diagrams showing the overlapping of the peaks of the short (35–80 nt) single-stranded cfDNA presented by Snyder et al. (15) and Burnham et al. (23) on the same (F) and antisense (G) strands of G4-seq peaks (left) and putative G4 sequences identified by the *quadparser* algorithm (right).

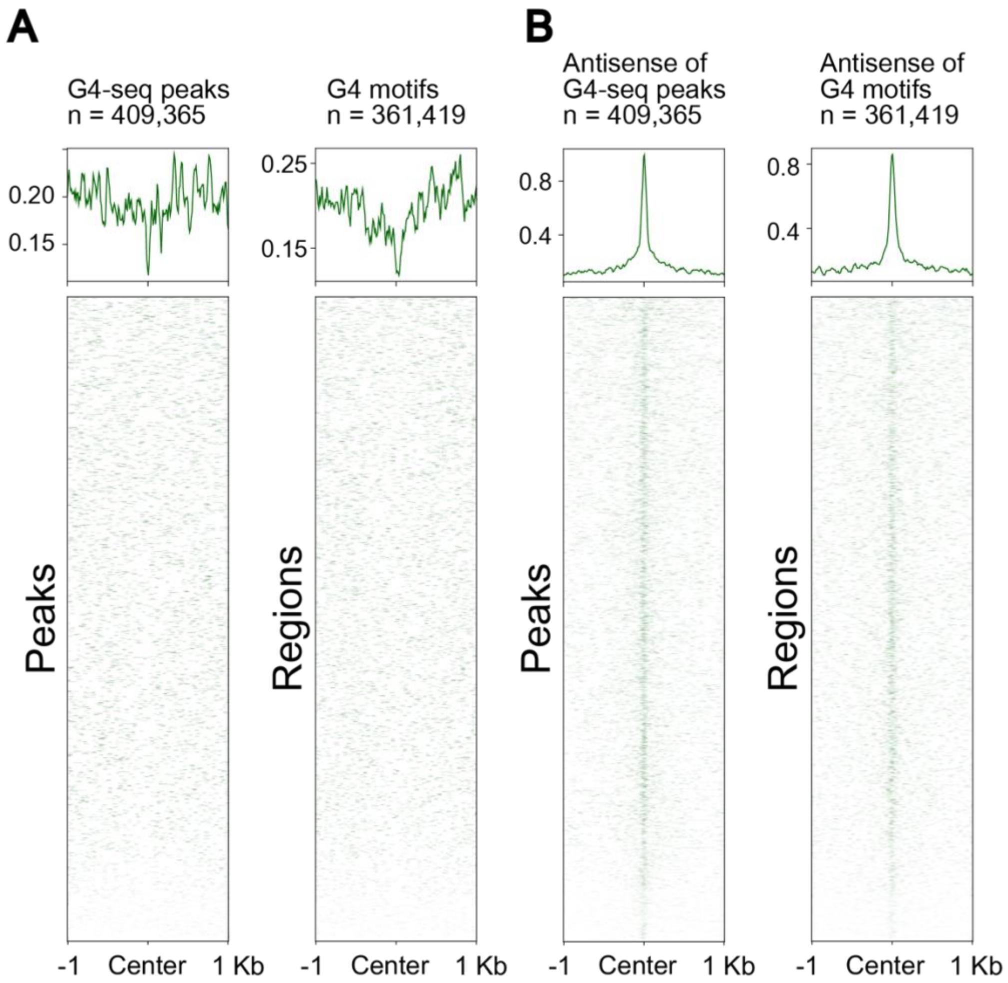

(Continues on the next page)

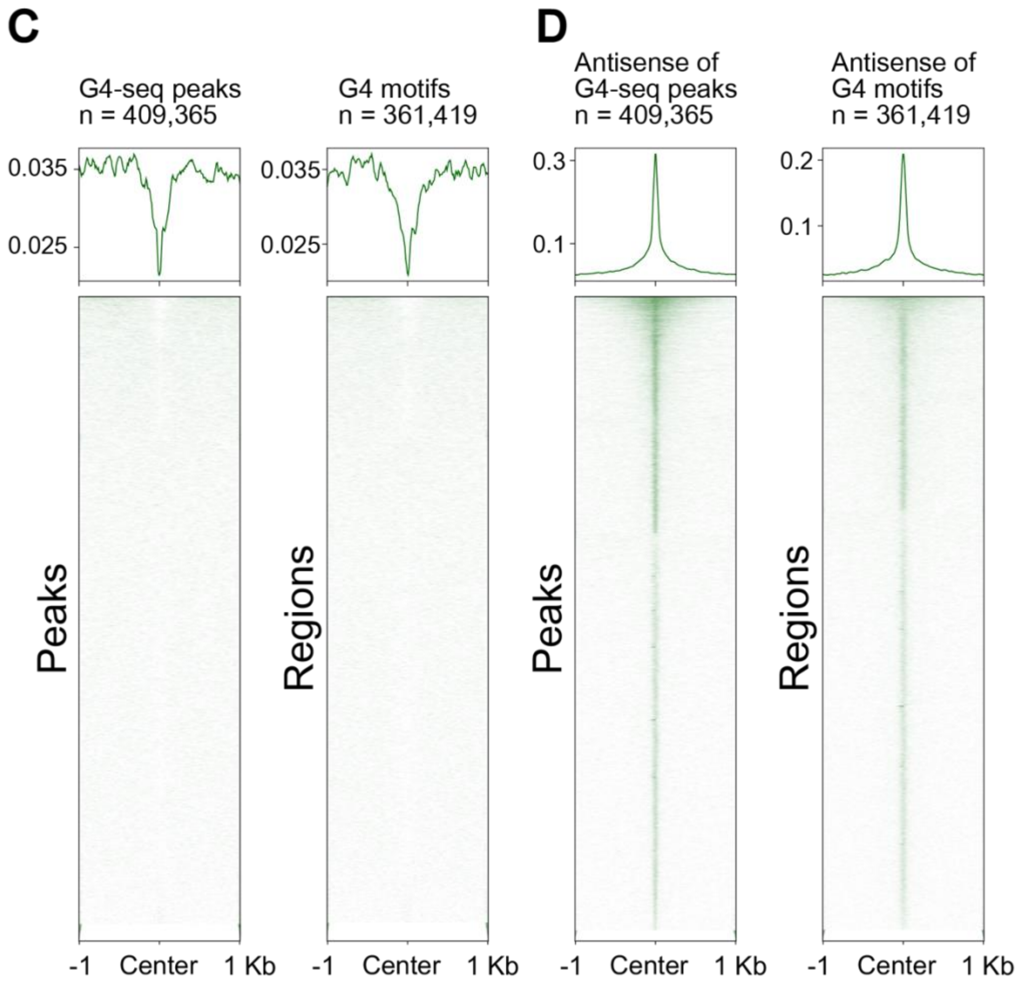

Figure S15. Enriched C3D reads on the G4 structure with different library preparation methods.

The aggregation plots for the C3D reads obtained with the libraries prepared by Gansauge et al. (2013) (20) (A and B) and Gansauge et al. (2017) (21) (C and D) were drawn using the same (A and C) and antisense (B and D) strands of G4-seq peaks and putative G4 sequences defined by the *quadparser* algorithm.

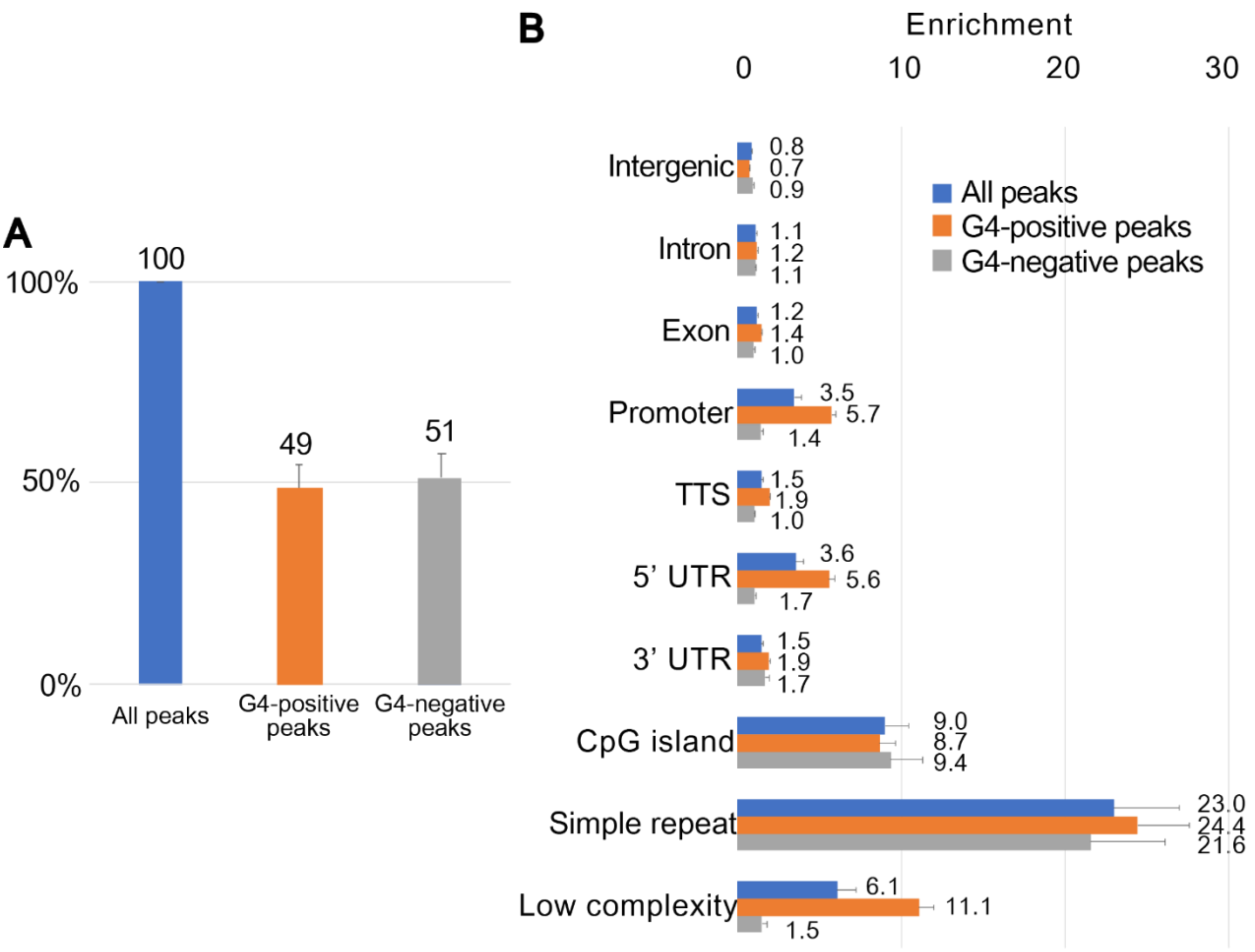

Figure S16. C3D peaks without antisense G4 structures are less enriched in the regulatory regions.

A. The fractions of C3D peaks overlapping with either G4-seq peaks or the G4 motif (G4-positive peaks) and those overlapping neither (G4-negative peaks) are shown. Average values for the five donors are calculated.

B. Enrichment analyses for all C3D peaks (same as Fig. 5B), G4-positive peaks, and G4-negative peaks were performed as described in Fig. 5B.

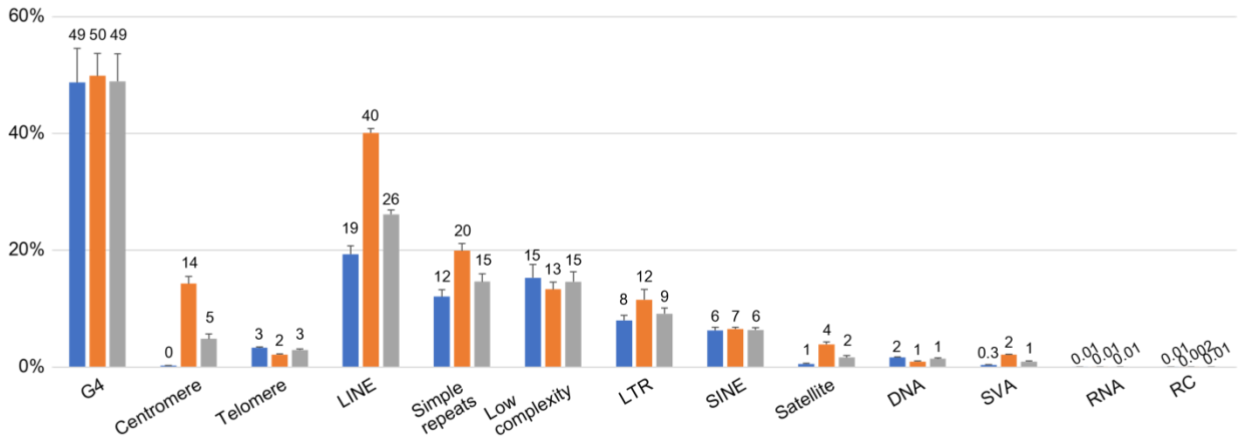

Figure S17. Colocalization of C3D peaks and repetitive sequences.

The fractions of C3D peaks overlapping indicated genomic features are shown as percentiles. Fractions calculated using only uniquely mapped reads are colored blue. Those with multimap reads are orange. Distributions of entire peaks are shown in gray. G4 indicates combined genomic regions overlapping either G4-seq peaks or G4 motifs. Centromere means a centromeric region defined using hg38 coordinates. Telomere indicates regions containing the TTAGGG motif. Other repetitive sequences defined in RepeatMasker were also indicated (Smi AFA 1996-2010). The average values of the five donors are shown.

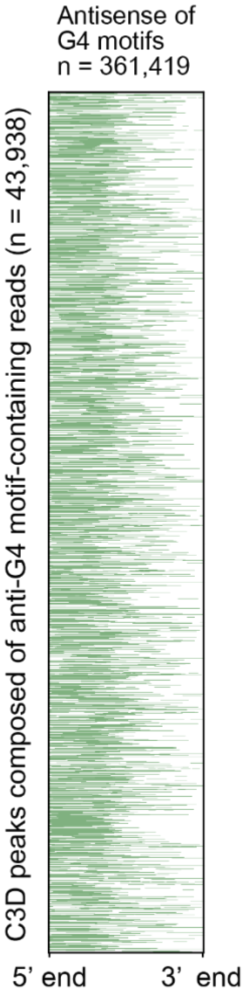

Figure S18. The antisense strands of G4 motifs are enriched at the 5′-end of C3D peaks.

Placement of antisense G4 motifs within the C3D peaks is shown in the heatmap. Only C3D peaks overlapping with G4 motifs were used to produce the image.

Supplementary Tables

Table S1. Summary of the library preparations and sequencing methods

Sample ID	DNA purification method*1	Library preparation					Sequencing Platform	Number of reads*3				Number of peaks		Percentages of reads in peaks*4	
		Pretreatment*2	Protocol	Input DNA (ng)	PCR cycles	Yield (pmol)		Total	Uniquely mapped	C3D	NPD	C3D	NPD	C3D	NPD
D1	PPIP	Heat	TACS-T4	6.6	5	105	MiSeq	643K	448K	194K	140K	NA	NA	NA	NA
D1	QIA	Heat	TACS-T4	3.9	5	26	MiSeq	351K	254K	NA	128K	NA	NA	NA	NA
D1	PPIP	Exo	TACS-T4	8.1	5	64	MiSeq	574K	443K	NA	158K	NA	NA	NA	NA
D1	PPIP		ThruPlex	6.6	5	126	MiSeq	660K	514K	NA	330K	NA	NA	NA	NA
D1	PPIP		TACS-T4	6.6	5	16	MiSeq	573K	356K	233K	62K	NA	NA	NA	NA
D1	PPIP		TACS-T4	8.6	5	16.9	HiSeq X	523M	266M	143M	72M	375K	493	25%	0.3%
D2	PPIP		TACS-T4	11	5	9.4	HiSeq X	531M	250M	176M	35M	284K	398	18%	0.2%
D3	PPIP		TACS-T4	11	5	2.2	HiSeq X	533M	255M	205M	25M	250K	342	21%	0.1%
D4	PPIP		TACS-T4	12	5	3.3	HiSeq X	544M	264M	184M	45M	186K	337	15%	0.2%
D5	PPIP		TACS-T4	8.6	5	8.6	HiSeq X	536M	193M	114M	41M	261K	365	25%	0.2%
D1	PPIP	Heat	TACS-T4	21	10	580	Miseq	1.6M	1.1M	402K	414K	NA	NA	NA	NA
D1	PPIP	Heat	Gansauge et al. (2013) (20)	21	16	2.2	Miseq	0.76M	0.42M	223K	93K	NA	NA	NA	NA
D1	PPIP	Heat	Gansauge et al. (2017) (21)	21	13	88	Miseq	1.3M	0.93M	525K	225K	NA	NA	NA	NA

*1 "PPIP" means that the procedure sequentially performed proteinase K treatment, phenol, and phenol-chloroform extractions, and isopropanol precipitation, as described in the Materials and Methods section. "QIA" means QIAamp Circulating Nucleic Acid Kit (Qiagen).

*2 "Heat" denotes heat denaturation. "Exo" means exonuclease (exonuclease I and RecJf) treatment.

*3 K and M denote thousands and millions, respectively.

*4 Percentages of C3D and NPD reads that overlap with the C3D and NPD peaks, respectively.

NA denotes "Not Applicable".

Table S2. The number of reads and peaks calculated from two publicly available single-stranded cfDNA datasets

Dataset name	DNA purification method*1	Sequencing		Number of reads*2				Number of peaks		Percentage of reads in peaks*3	
		Library preparation method	Platform	Total	Uniquely mapped	Short*4	Nucleosome*4	Short*4	Nucleosome*4	Short*4	Nucleosome*4
Snyder et al. (15)	QIA	Gansauge et al. (2013) (20)	HiSeq 2000 and NextSeq 500	638M	473M	80M	249M	1,008	774	0.5%	0.3%
Burnham et al. (23)	QIA	Gansauge et al. (2013) (20)	MiSeq and HiSeq	207M	154M	19M	94M	963	379	0.4%	0.3%
This study (Mean value)	PPIP	TACS-T4	HiSeq X	533M	246M	165M	73M	271,628	387	21%	0.2%

*1 “PPIP” means the procedure sequentially performed proteinase K treatment, phenol, phenol-chloroform extractions, and isopropanol precipitation, as described in the Materials and Methods section. “QIA” means QIAamp Circulating Nucleic Acid Kit (Qiagen).
*2 M denotes million.
*3 Percentage of reads overlapping with the peaks.
*4 “Short” means 35–80 nt reads. “Nucleosome” indicates the nucleosome size range (120–180 nt) reads from previous studies. The mean values of C3D and NPD reads are shown in this study.

Tables S3, S4, and S5 have been supplied in Additional file 2, 3, and 4, respectively.

Table S6. Numbers of uniquely mapped reads and multiply mapped reads

Sample ID	Uniquely mapped reads			Uniquely mapped peaks		Multiple mapped reads			Multiple mapped peaks		Fractions of uniquely mapped reads (%)		
	All	C3D	NPD	C3D	NPD	All	C3D	NPD	C3D	NPD	All	C3D	NPD
D1	266M	143M	72M	375K	493	63M	39M	12M	147K	5,740	81%	79%	86%
D2	250M	176M	35M	284K	398	63M	46M	6.1M	137K	2,985	80%	79%	85%
D3	255M	205M	25M	250K	342	69M	56M	4.5M	128K	2,661	79%	78%	85%
D4	264M	184M	45M	186K	337	66M	49M	7.6M	119K	2,734	80%	79%	86%
D5	193M	114M	41M	261K	365	51M	33M	7.2M	108K	2,924	79%	77%	85%

*** K and M denote thousands and millions, respectively.**

Table S7. Blood samples used in the current study

Sample ID	Sample type	Age (years), Sex	Supplier*1
D1	Healthy donor	32, Male	
D2	Healthy donor	30, Male	
D3	Healthy donor	43, Male	
D4	Healthy donor	44, Male	
D5	Healthy donor	35, Male	
P1	Purchased plasma	21, Female	BIOPREDIC
P2	Purchased plasma	48, Male	BIOPREDIC
P3	Purchased plasma	52, Male	BIOPREDIC
S1	Purchased serum	61, Female	CTLS
S2	Purchased serum	29, Male	Cosmo bio

*1 BIOPREDIC, CTLS, Cosmo bio indicate that we obtained these samples from BIOPREDIC (Rennes, France), Clinical Trials Laboratory Services (London, UK), Cosmo Bio (Tokyo, Japan).

Table S8. Oligonucleotides used in the current study

Name	Used for	Nucleotide sequence and chemical modifications
PA-Truseq Index-dSp-P	TACS ligation	5'-[phosphate] AGATCGGAAGAGCACACGTCTGAACTCCAG [dSpacer] CAC [phosphate]-3'
TruseqUniv-T	Complementary strand synthesis	5'-TCTTTCCCTACACGACGCTCTTCCGATCTT-3'
PCR-Univ	Indexing and amplification	5'-AATGATACGGCGACCACCGAGATCTACACTCTTTCCCTACACGAC-3'
PCR-Index-X[*1]	Indexing and amplification	5'-CAAGCAGAAGACGGCATACGAGAT [Index Sequence] GTGACTGGAGTTCAGACGT-3'
N [Number][*2]	Size marker and model substrate	5'-N[Number]-3'
rA40[*3]	Control for RNase digestion	5'-[rA]40-3'
200 bp-forward	PCR primer for dsDNA control	5'-CGACCTTTCTGTGGTGAA-3'
200 bp-reverse	PCR primer for dsDNA control	5'-CACATGTCGCGGTGGTTA-3'

*1 "X" is the index number, i.e., a specific hexamer was inserted in the position "[Index Sequence]" for indexing sequences (see Table S9).

*2 N denotes an equimolar mixture of A, C, G, and T. "[Number]" refers to the nucleotide length. In the current study, stretches of 40, 60, 80, 100, 120, 140, and 160 nt were used.

*3 [rA] denotes an adenosine residue.

Table S9. Indexing sequences

Index number[*1]	Index sequences[*2]
1	5'-CGTGAT-3'
2	5'-ACATCG-3'
3	5'-ACATCG-3'
4	5'-TGGTCA-3'
5	5'-CACTGT-3'

*1 This is the same as “X” used in Table S8.
*2 This is the “[Index Sequence]” used in Table S8.

Table S10. Sources of publicly available data used in the current study

Data name	Reference	Accession number (Database)
FANTOM5 enhancers and promoters	Andersson et al. (28)	
RefSeq genes	O'Leary et al. (30)	-
ATAC-seq of GM12878	Buenrostro et al. (31)	GSE47753 (NCBI GEO)
ENCODE TFBS cluster version 3	Gerstein et al. (32)<br>Rosenbloom et al. (33)	-
ChIP-Atlas	Oki et al. (34)	-
G4-seq reads and peaks	Chambers et al. (35)	GSE63874 (NCBI GEO)
CpGi from UCSC table browser	Karolchik et al. (36)	-
Snyder et al. (15)	Snyder et al. (15)	GSE71378 (NCBI GEO)
Burnham et al. (23)	Burnham et al. (23)	PJRNA306662 (NCBI SRA)
RepeatMasker from UCSC table browser	Smit et al. (37)	-

Table S11. Files of the ENCODE datasets downloaded from the UCSC Table Browser

Experiment type	Cell line	BigWig file	BED file
DNase-seq	GM12878	wgEncodeOpenChromDnaseGm12878Sig.bigWig	wgEncodeOpenChromDnaseGm12878Pk.narrowPeak
CTCF ChIP-seq	GM12878	wgEncodeUwTfbsGm12878CtcfStdRawRep1.bigWig	wgEncodeUwTfbsGm12878CtcfStdPkRep1.narrowPeak.gz
MNase-seq	GM12878	wgEncodeSydhNsomeGm12878Sig.bigWig	-

Supplementary Methods

Preparation of model dsDNA fragments

We prepared a model double-stranded DNA fragment (DNA200). A 50-µL reaction containing 1× PrimeStar Max (Takara Bio Inc.), 0.2 µM each of 200 bp-forward and 200 bp-reverse (Table S7), and 5 ng of lambda DNA was prepared. Then, PCR amplification was performed with the following conditions: initial denaturation at 95°C for 1 min; 35 cycles of 2-step incubation at 95°C for 15 sec and 72°C for 30 sec; final extension at 72°C for 5 min. Following the manufacturer's instructions, the amplified DNA was purified with AMPure XP (Beckman Coulter, Brea, CA, USA).

Fig. 1C and D

A model mixture was prepared by mixing equimolar amounts of the synthetic DNA, N40, N80, N120, and DNA200 at 0.1 µM in 10 mM Tris-HCl (pH 8.0). Next, 5 µL of purified cfDNA, which was equivalent to 0.25 mL of plasma, was supplemented with 0.2 µL of the control mixture, and subjected to 3′-terminal fluorescent labeling and analyzed as described in Materials and Methods.

Fig. 1E and F

A model mixture was prepared by mixing equimolar amounts of the synthetic DNA N60, N80, N100, N120, N140, N160, synthetic RNA rA40 (see Table S7), and DNA200 at 0.05 µM in 10 mM Tris-HCl (pH 8.0). In 2 µL of the mixture or 10 µL of cfDNA extracted from 1 mL plasma, 1.2 µL 10× TACS buffer (500 mM HEPES-KOH (pH 7.5), 50 mM $MgCl_2$, and 5% (v/v) Triton-X100), and 1.2 µL 10× phosphate buffered saline (PBS) were supplemented, and the reaction volume was adjusted to 12 µL. Nuclease digestion was started with the addition of 15 units of RNase If (New England Biolabs; NEB, Ipswich, MA, USA), 1 unit of RQ1 RNase-Free DNase (Promega, Fitchburg, WI, USA), or a mixture containing both six units of exonuclease I and ten units of RecJf (NEB). The reaction mixture was incubated at 37°C for 30 min, diluted in 1 mL PBS, and purified again using the PPIP method. The purified DNA was analyzed as described in the Materials and Methods.

Fig. 1G, H and Figure S1D

First, a tube containing 0.5 mL of plasma was supplemented with 5 µL of 1 M $MgCl_2$. If indicated, 1 µL of the model mixture, as used in Fig. 1D, was spiked into the plasma. Then, 50 units of RNase If (NEB), 20 units of RQ1 RNase-Free DNase (Promega), or a mixture

containing 20 units of exonuclease I and 30 units of RecJf were added. The reaction was incubated at 37°C for 30 min and was then purified using the PPIP method. Incubation time was extended up to 16 hours as described in Fig. S1D. The purified cfDNA was analyzed via denaturing gel electrophoresis, as described in Materials and Methods.

Figure S1B, S1C, and S2B

A control mixture was prepared by mixing N40, N80, N120, and DNA200 at 0.1 μM in 10 mM Tris-HCl (pH 8.0). To 500 μL of plasma or serum, 0.4 μL of the control mixture was added, and the cfDNA was purified, as described in Materials and Methods. The purified DNA was analyzed as described in the Materials and Methods. The amounts of the supernatants or pellets after ultracentrifugation equivalent to 500 μL of plasma were used for the results presented in Figure S2B.

Fig. 2E and Figure S4D

Plasma (0.5 mL), 1 M $MgCl_2$ (5 μL), exonuclease I (1 μL), and RefJf (1 μL) were combined and incubated at 37°C for 30 min. Then, cfDNA was purified as described in the Materials and Methods section. The purified cfDNA was then used for the TACS-T4 scheme, as described in Materials and Methods.

Fig. 2G and Figure S4F

For the library "without heat denaturation," TACS ligation was performed without any pretreatment of cfDNA, as follows. A reaction was prepared by mixing 10 μL of cfDNA extracted from 0.5 mL plasma, 2.5 μL of 10× TACS Buffer, 10 μL of 50% (w/v) PEG, 1 μL of 10 μM PA-TruSeqIndex-dSp-P (Table S8), 1 μL of 10 mM ATP, 1 μL of TdT, and 1 μL of 2 mg/mL TS 2126 RNA ligase in a 25 μL volume and sequentially incubated at 37°C for 30 min, 65°C for 2 h, and 95°C for 5 min. After this step, library preparation was performed as described in the Materials and Methods section.

Expression and purification of TS2126 RNA ligase

The previously used plasmid encoding TS2126 RNA ligase ((38); Addgene #119941) was modified with inverse PCR so that the recombinant protein had a Strep-Tag at its carboxyl-terminal (Addgene #159350). The plasmid was then used to transform T7 Express *E. coli* (NEB). Transformants were inoculated into 3 mL of 2× YT medium (1.6% [w/v] Bacto Tryptone, 1% [w/v] Bacto yeast extract, 0.5% glucose, and 0.5% [w/v] NaCl) supplemented

with 50 μg/mL kanamycin. They were grown at 37°C with continuous shaking at 250 rpm for 16–18 h. The cultures were diluted in 1 L of 2× YT medium containing 50 μg/mL of kanamycin and incubated at 37°C with continuous shaking at 120 rpm for 4–5 h. Protein expression was induced by the addition of isopropyl-β-D-thiogalactopyranoside (IPTG) powder (Nacalai Tesque, Kyoto, Japan) at a final concentration of 1 mM, followed by further incubation at 37°C with continuous shaking at 120 rpm for 4 h. Cells were collected via centrifugation, suspended in 20 mL of HisTrap Buffer A (50 mM sodium phosphate, pH 7.0, 100 mM NaCl, and 10 mM imidazole), and then lysed via sonication using a Digital Sonifier (Branson, Danbury, CT, USA). The cell debris was removed by centrifugation at 10,000 × *g* for 10 min, followed by filtration using 32-mm Acrodisc syringe filters with Supor Membrane (0.45-μm) (Pall, Port Washington, NY, USA). The cleared cell lysate was loaded onto the AKTA start chromatography system (GE Healthcare, Chicago, IL, USA) equipped with a HisTrap HP column (5-mL, GE Healthcare) using an equipped template for affinity chromatography. The column was equilibrated and washed with HisTrap Buffer A, and the purified protein was eluted with HisTrap Buffer B (50 mM sodium phosphate, pH 7.0, 100 mM NaCl, and 200 mM imidazole). Fractions containing the target protein were combined, diluted 20-fold with Strep buffer A (100 mM Tris-HCl, pH 8.0, 100 mM NaCl, and 1 mM EDTA), and further purified using the AKTA start system equipped with a StrepTrap HP column (5 mL, GE Healthcare). StrepTrap purification was performed using an equipped template for affinity chromatography with Strep Buffer A and Strep Buffer B (100 mM Tris-HCl, pH 8.0, 100 mM NaCl, 1 mM EDTA, and 2.5 mM d-Desthiobiotin). Fractions containing the target protein were combined and subjected to ultra-filtration on an Amicon-4 30K device (Millipore, Burlington, MA, USA) to exchange the buffer with 2× storage buffer (100 mM Tris-HCl [pH 8.0], 150 mM NaCl, 1 mM EDTA) and concentrate the protein. Protein concentration was determined using the Protein Assay CBB Solution (Nacalai Tesque). After adjusting the protein concentration to 4 mg/mL, the protein solution was combined with an equal volume of glycerol and stored at -20°C until use.

Expression and purification of recombinant human aprataxin

DNA fragments encoding human aprataxin (UniProt#Q7Z2E3) were chemically synthesized by Eurofins Genomics (Tokyo, Japan) with codon optimization for *Escherichia coli*. Recognition sequences for BamHI and EcoRI were introduced at the 5' and 3' ends of the protein-coding sequence, respectively. The fragment was subcloned into the BamHI–EcoRI site of pColdI (Takara Bio Inc.). The obtained plasmid was then used to transform *E. coli*

BL21 (Takara Bio Inc.). The transformants were inoculated into 3 mL of 2× YT medium containing 50 μg/mL of carbenicillin. They were grown at 37°C with continuous shaking at 250 rpm for 16–18 h. Next, the cultures were diluted in 1 L of 2× YT medium containing 50 μg/mL of carbenicillin and grown with shaking at 37°C for 4–5 h. The culture was then cooled to 16°C in ice-cold water for 30 min. Protein expression was induced by the addition of IPTG powder to obtain a final concentration of 1 mM. The culture was then incubated with shaking at 16°C for at least 16 h. The first purification using the HisTrap HP column was performed as described for the purification of TS2126 RNA ligase. Fractions containing the target protein were combined, diluted 20-fold with heparin buffer A (50 mM Tris-HCl, pH 8.0), and further purified using the AKTA start system equipped with a HiTrap Heparin HP column (GE Healthcare). The second purification was performed using an equipped template for ion-exchange chromatography with heparin buffers A and B (50 mM Tris-HCl [pH 8.0], and 1 M NaCl). The subsequent buffer exchange was performed using the Amicon-4 30K device, as described for the purification of TS2126 RNA ligase. After adjusting the protein concentration to 2 mg/mL, the solution was combined with an equal volume of glycerol and stored at -20°C until use.

Fig. 3

First, bowtie2 was used to map the sequenced reads on the human reference genome, including those for nuclear and mitochondrial sequences. The unmappable reads of the human genome were compared with the bacterial genome by using NCBI BLAST (39) and reference databases comprising viral, bacterial, and fungal genome sequences (downloaded from NCBI ftp://ftp.ncbi.nih.gov/genomes on April 17, 2019), as described by Vlaminck et al. (40).

Fig. 4C

The normalized read coverage of C3D peaks for the five donors (Table S1) was converted to a matrix with deepTools multiBigwigSummary and plotted with plotCorrelation (41).

Figure S7B

GO Enrichment analysis was performed by PANTHER (42), and GOslim was used as a GO term set. Fisher’s exact test was performed against all reference genes using R.

Pipelines for bioinformatic analyses

The commands used in the bioinformatic analyses are shown in Supplementary Information S3, with flowcharts of analytical pipelines.

Supplementary Information

Supplementary Information S1. Nucleotide sequence of a gene encoding codon-optimized TS2126 RNA ligase with a Strep-Tag.

Recognition sequences for BamHI and EcoRI are underlined.
The sequence encoding the Strep-Tag is shown in bold.

GGATCCATGAGCTCACTGGCTCCGTGGCGTACGACGAGCTGGAGTCCGCTGGGCTCTCCGCC
AAGTTTAGAGGATGCTTTGCGTCTTGCGCGCACAACTCGCGCATTCGCAGTCCGCCGCGATG
GTGAAGGTCGCGCATTGGTTACCTACCTGTATGGCACTCCCGAGCTGTTCTCCCTGCCGGGC
GCGCGTGAATTGCGTGGTATCGTGTATCGCGAGGAGGATGGCACCGTGCTGAGCCGTCCGTT
TCACAAATTCTTCAACTTTGGAGAACCGTTAGCTCCGGGTGAAGAGGCCTTTAAAGCATTTC
GCGATTCGATGGTGCCCCTGTTTGTCGCCGAGAAAGTGGATGGCTACCTGGCACAAGCGTAC
CTGGATGGTGGGGAGTTGCGTTTTGCCTCTCGGCATAGCCTTAATCCGCCACTTGTGGGTGC
GTTGCTGCGCAAAGCCGTCGATGAAGAAGCGATGGCGCGTCTGGGAAAACTCTTAGCTGCGG
AAGGCGGGCGTTGGACGGCCCTGTTAGAAGTGGTTGATCCGGAAGCGCCGGTCATGGTACCG
TATCAGGAACCAGGCGTGTATCTGCTGGCCCTCCGTTCGATTGGTGAAGGGCACTATCTTCT
GCCTGGGGTACATTTCCCGCTGCCTGAAGCCCTGCGTTACGTTCGGTGGGAACCACGCATGG
ACTTTGACCCTCATCGCTTTCGCGGTGAAATTCGCGACCTCCAAGGCGTAGAGGGCTACGTG
GTTACCGATGGTGCGGAGTTTGTCAAGTTCAAAACCGGCTGGGCGTTTCGGTTAGCGCGCTT
CCTGATGGACCCCGAAGGGGTGTTCCTGGAAGCCTATGCGGAAGATCGGCTGGACGACCTGG
TGGGTGCCTTGGCGGGCCGCGAGGACCTCCTGCGTGCGGTTGCGCGTGCGCAGGATTACCTG
GCAGGACTCTATGGTGAAGCAGTTGGAGCTGGCGATGCCTTACGCCGTATGGGCCTTCCGCG
CAAGGAAGCATGGGCGCGTGTACAGGAAGAGGCCGGTCGTTGGGGCGGCTTTGCCCCTGCGT
ATGCTCGCGCAGCAATGGCCGCATATGAAGGCGGCGAAGCCCGCGAAGCGTTTCTGGTCGAA
CTGCGCAAACGCTCCGCTCGTAAAGCTCTGGAAGCTCTGCACTTGTTCCCACGCGTTGGTGG
GGAATTACGCGGTGAATTC**TGGAGCCATCCGCAGTTTGAAAAA**

Supplementary Information S2. Nucleotide sequence of a gene encoding codon-optimized human aprataxin.

Recognition sequences for BamHI and EcoRI are underlined.

GGATCCATGAGCAATGTGAATCTCAGCGTAAGCGATTTCTGGCGCGTGATGATGCGTGTCTG
TTGGTTGGTGCGGCAGGATTCACGCCATCAACGCATACGTCTGCCTCATTTGGAGGCTGTAG
TGATTGGCAGAGGTCCGGAAACGAAGATTACCGACAAAAAATGCAGTCGTCAGCAAGTCCAA
CTGAAAGCGGAATGCAACAAAGGGTACGTTAAGGTGAAACAGGTAGGTGTCAACCCCACGTC
CATTGATTCGGTGGTGATTGGGAAAGATCAGGAGGTAAAACTCCAACCAGGTCAGGTTCTGC
ACATGGTGAATGAGCTGTATCCGTATATCGTCGAGTTCGAAGAAGAGGCCAAAAATCCGGGC
CTCGAAACCCATCGCAAACGCAAACGTAGTGGCAACTCCGACTCTATCGAACGCGATGCAGC
CCAAGAAGCGGAAGCAGGTACTGGTCTTGAACCAGGCTCAAATAGTGGTCAGTGTTCGGTTC
CGCTCAAGAAAGGCAAGGATGCGCCGATCAAGAAAGAGAGTCTTGGCCATTGGTCCCAAGGC
TTGAAAATCTCCATGCAAGATCCGAAGATGCAGGTCTATAAAGACGAACAGGTTGTCGTGAT
CAAAGACAAATACCCGAAAGCGCGTTATCATTGGCTGGTTTTGCCCTGGACCAGCATTAGCT
CTCTGAAAGCCGTGGCACGTGAACACCTGGAGTTACTGAAACACATGCACACTGTAGGGGAA
AAAGTGATCGTTGACTTTGCGGGATCTTCGAAACTGCGCTTTCGCCTAGGCTATCACGCCAT
TCCGAGCATGTCACACGTGCATCTGCACGTTATTAGCCAGGATTTTGATTCACCGTGTCTGA
AGAACAAAAAGCATTGGAACAGCTTTAACACGGAATACTTCCTGGAATCTCAAGCTGTCATC
GAAATGGTGCAGGAAGCTGGTCGTGTTACCGTTCGAGACGGAATGCCTGAGCTGCTTAAATT
ACCGTTACGGTGCCATGAATGCCAGCAGTTACTGCCTTCGATTCCACAGCTGAAAGAACATC
TGCGTAAACATTGGACACAGGAATTC

Supplementary Information S3.

Flowcharts for bioinformatic analyses

Trimming and mapping

FASTQ

fastp: Remove low-quality reads
$ fastp (no option)

FASTQ, filtered

seqkit subseq : Trim nucleobases added in TACS-T4
$ seqkit subseq -r 3:-2 #Read 1
-r 2:-3 #Read 2

FASTQ, trimmed

bowtie2: Map reads in paired-end mode
$ bowtie2 -q -p 10 -x *human_hg19*

BAM

samtools view: Extract the reads whether mapped to nuclear genome, mapped to mitochondria, or unmapped
$ samtools view -f 2 | awk '$3!="LAMBDA"' |awk '$3!="chrM"' #nuclear genome
$ samtools view chrM #mitochondria
$ samtools view -f 4 #unmapped

BAM

awk: Extract uniquely mapped reads
$ awk '{if(NR%2)ORS="~";else ORS="¥n";print}' | grep -v ".*XS.*XS" |/
$ awk -F "~" '{ print $1 "¥n" $2 }'

BAM, uniquely mapped

Separation of mapped reads and peak calling

BAM, uniquely mapped

awk: Separate the alignments by the fragment sizes

```
$ awk '$9!=0' | awk '{print sqrt($9^2) , $0}' |/
 '$1 >= 35' | awk '$1 <= 75' | cut -f 2- -d " "       #C3D reads
 '$1 >= 147' | awk '$1 <= 190' | cut -f 2- -d " "   #NPD reads
```

BAM, extracted by fragment size

samtools view: Separate the alignments by the mapped reference genome strand

```
$ samtools view "-f 128 -F 16" or "-f 80"          #reads mapped on top strand
$ samtools view "-f 64 -F 16" or "-f 144"          #reads mapped on bottom strand
```

BAM, separated with mapped strand

MACS2: Peak call

$ macs2 callpeak -f BAMPE -g hs -q 0.01

$ cat *peak_top.bed peak_bottom.bed* > *peak_merged.bed*

BED

Calculation of the read coverages

BAM

↓

bedtools genomecov: Calculate coverage of each fragments
$ bedtools genomecov -bga -pc

BEDGRAPH

↓

bedGraphToBigWig: Convert bedGraph to bigWig
$ bedGraphToBigWig (no option)

BIGWIG

Size distribution plot
(Fig. 2, 3, Figure S5F-H, S6A)

BAM

Calculation of size distribution from TLEN column in SAM format:

```
$samtools view |cut -f 9 |tr -d "-” |/
$awk '{ v[$0]++ } END { for ( k in v ) print k "¥t" v[k]/2 }’ |/
$sort -n |awk '$1 <= 500'
```

TSV

Scatter plot made in Excel

PDF

BLAST for unmapped reads
(Fig. 3)

BAM, unmapped to nuclear or mitochondrial genome

SamtoFastq (picard tools): Make fastq from bam
$ java -jar picard.jar SamToFastq (no option)

FASTQ

BBMerge (BBTools): Merge paired fastq to single-end like fastq
$ bbmerge-auto.sh (no option)

FASTQ, merged

fastp: Quality filtering of merged reads
$ fastp (no option)

FASTQ, filtered

fastq_to_fasta (fastx_toolkit) : Convert fastq to fasta format
$ fastq_to_fasta (no option)

FASTA

BLASTn: Realign of unmapped reads to microbial sequences
$ blastn -db *blastdb** -outfmt "17 SQ" /
-reward 1 -penalty -3 -word_size 12 -gapopen 5 -gapextend 2 -evalue 1E-4 /
-perc_identity 90 -culling_limit 2

**blastdb* ;of blastdb_bacteria, blastdb_virus, blastdb_fungi
Source of the database sequences were downloaded from NCBI database (ftp://ftp.ncbi.nih.gov/genomes).

BAM

Visualization with genome browser
(Fig. 4A, 6A and 7C)

BIGWIG,
BED

↓

UCSC genome browser: Visualize the coverage and the peak tracks
Track Hub URL: http://itolab.med.kyushu-u.ac.jp/hisano/tracks_for_ucsc/hub.txt

PDF

Comparing the coverage of C3D peaks among the donors
(Fig. 4C)

BIGWIG

↓

multiBigwigSummary (deeptools): Calculate average coverage in equally sized bins
multiBigwigSummary bins --binSize=100

NPZ, matrix file

↓

plotCorrelation (deeptools): Visualize the coverage correlation in bins
plotCorrelation --corMethod pearson --skipZeros --removeOutliers ¥
--whatToPlot scatterplot --xRange 0 500 --yRange 0 500

PDF

Aggregation plot and heatmap
(Fig. 4-7, Figure S7-9, S11, S13-15)

BED, regions **BIGWIG**

Computematrix (deeptools): Make matrix files for plot

```
$ computeMatrix reference-point --referencePoint center /
  --upstream 1000 --downstream 1000        #plot around the peak center
$ computeMatrix scale-regions ¥
  --upstream 1000 --downstream 1000        #regard the region range
```

TXT, matrix file

plotHeatmap (deeptools): Plot heatmaps

```
$ plotHeatmap
  --missingDataColor 'white' /
  --outFileSortedRegions
```

PDF

HOMER peak annotation
(Fig. 5A, 5B, Figure S7D, S16)

BED

annotatePeaks (HOMER): Annotate peaks to known functional elements
$ annotatePeaks hg19 -annStats

TSV

Bar chart made in Excel

PDF

GO enrichment analysis
(Figure S7B)

TSV, result of HOMER annotation

↓ Gene ID list of the C3D peaks with annotation "5' UTR " or "promoter" (n = 7,634) was selected according to the HOMER annotation.

TXT, Gene ID list

↓ **Panther gene list analysis** : Functional classification of genes
The analysis performed on website (http://pantherdb.org) and the result was downloaded as text file.
Total reference gene list supplied from the Panther website (n = 20,851).

TXT

↓ Fold enrichment was calculated against total reference genes in Excel

R: Fisher exact test of GO in C3D-regulated genes against reference genes.
fisher.test() (no option)

PDF

Venn diagram, overlap test of the peaks
(Fig. 6, 7, Figure S6-10, S14)

BED, regions

BED

ChIPpeakAnno: Calculate and make venn diagram of overlapping peaks
findOverlapsOfPeaks() (no option)
makeVennDiagram() (no option)

permTest (regioneR): Permutation test of overlapping peaks

```
permTest(ntimes=1000, count.once=TRUE, genome="hg19",
         force.parallel=FALSE, randomize.function=randomizeRegions,
         evaluate.function=numOverlaps, verbose=FALSE)
```

PDF

Calculation of the base composition of the peaks
(Fig. 7B, Figure S12)

BAM

bedtools bamtobed: Acquire genomic coordinates from paired alignments
$ bedtools bamtobed -bedpe |awk 'BEGIN{OFS="¥t"} {print($1, $2, $6)}'

BED

bedtools getfasta: Extract sequences from genome coordinates
$ bedtools getfasta (no option)

FASTA

seqkit fx2tab: Calculate base contents of each fragments
$ seqkit fx2tab -g -G -B A -B T -B C -B G

TSV

ggplot2: visualize histogram of base contents
$ ggplot(aes(x = **base*)) + geom_histogram(binwidth = 1)
**base*; A, T, C, G, GC-skew

Calculation of average base content in Excel

PDF

Calculation of the peak strand bias of the peaks
(Figure S12A)

BED,
C3D

BIGWIG,
C3D separated by mapped strand

Computematrix (deeptools): Make matrix files of coverage
$ computeMatrix scale-regions

TXT, matrix file

Strand bias of each C3D peak was calculated in Excel
strand_bias = (*Top* - *Bottom*) / (*Top* + *Bottom*)
Top; average coverages of the reads mapped on the top strand within C3D peak
Bottom; average coverages of the reads mapped on the bottom strand within C3D peak

TSV

ggplot2: Visualize histogram of base contents
$ ggplot(aes(x = strand_bias)) + geom_histogram(binwidth = 1)

PDF

G4motif calculated by the Quadparser algorithm
(Fig. 7, Figure S14-16)

FASTA, whole genome

↓

fastaregexfinder: Search a fasta file for matches to a regex and return a bed file
(https://github.com/dariober/bioinformatics-cafe/blob/master/fastaRegexFinder/)
fastaRegexFinder.py -r '([gG]{3,}¥w{1,7}){3,}[gG]{3,}'

BED

Random coordinates from hg19 genome
(Figure 7, S9, S10, S12)

FASTA, whole genome

↓

bedtools random: Generate a random set of intervals from reference genome
$ bedtools random -seed 11 -l 55 -n 100000

BED
